# Glucocorticoid receptor regulates PD-L1 and MHC-I in pancreatic cancer cells to promote immune evasion and immunotherapy resistance

**DOI:** 10.1038/s41467-021-27349-7

**Published:** 2021-12-06

**Authors:** Yalan Deng, Xianghou Xia, Yang Zhao, Zilong Zhao, Consuelo Martinez, Wenjuan Yin, Jun Yao, Qinglei Hang, Weiche Wu, Jie Zhang, Yang Yu, Weiya Xia, Fan Yao, Di Zhao, Yutong Sun, Haoqiang Ying, Mien-Chie Hung, Li Ma

**Affiliations:** 1 Department of Experimental Radiation Oncology, The University of Texas MD Anderson Cancer Center, Houston, TX 77030 USA; 2 Department of Molecular and Cellular Oncology, The University of Texas MD Anderson Cancer Center, Houston, TX 77030 USA; 3 Department of Breast Surgery, Zhejiang Cancer Hospital, Institute of Cancer and Basic Medicine, Chinese Academy of Sciences, 310022 Hangzhou, Zhejiang China; 4 Department of Pathology, Zhejiang Cancer Hospital, Institute of Cancer and Basic Medicine, Chinese Academy of Sciences, 310022 Hangzhou, Zhejiang China; 5 Hubei Hongshan Laboratory, College of Life Science and Technology, College of Biomedicine and Health, Huazhong Agricultural University, 430070 Wuhan, Hubei China; 6 The University of Texas MD Anderson UTHealth Graduate School of Biomedical Sciences, Houston, TX 77030 USA; 7 Graduate Institute of Biomedical Sciences and Center for Molecular Medicine, China Medical University, Taichung, 404 Taiwan; 8 Department of Biotechnology, Asia University, Taichung, 413 Taiwan

**Keywords:** Pancreatic cancer, Cancer immunotherapy

## Abstract

Despite unprecedented responses of some cancers to immune checkpoint blockade (ICB) therapies, the application of checkpoint inhibitors in pancreatic cancer has been unsuccessful. Glucocorticoids and glucocorticoid receptor (GR) signaling are long thought to suppress immunity by acting on immune cells. Here we demonstrate a previously undescribed tumor cell-intrinsic role for GR in activating PD-L1 expression and repressing the major histocompatibility complex class I (MHC-I) expression in pancreatic ductal adenocarcinoma (PDAC) cells through transcriptional regulation. In mouse models of PDAC, either tumor cell-specific depletion or pharmacologic inhibition of GR leads to PD-L1 downregulation and MHC-I upregulation in tumor cells, which in turn promotes the infiltration and activity of cytotoxic T cells, enhances anti-tumor immunity, and overcomes resistance to ICB therapy. In patients with PDAC, GR expression correlates with high PD-L1 expression, low MHC-I expression, and poor survival. Our results reveal GR signaling in cancer cells as a tumor-intrinsic mechanism of immunosuppression and suggest that therapeutic targeting of GR is a promising way to sensitize pancreatic cancer to immunotherapy.

## Abstract (web summary)

Glucocorticoids and glucocorticoid receptor (GR) signalling can suppress anti-tumour immunity. Here the authors show that GR activates PD-L1 expression and represses MHC-I expression in pancreatic cancer cells, while GR inhibition enhances anti-tumour immunity and sensitises the cancer cells to immunotherapy.

## Introduction

Pancreatic cancer, primarily pancreatic ductal adenocarcinoma (PDAC), is a leading cause of cancer-related mortality, with a 5-year survival rate of as low as 6% in the United States^1^. Unfortunately, only a small subset of patients diagnosed with PDAC present with localized and surgically resectable tumors^2^. Chemotherapy and radiotherapy are routinely employed in pancreatic cancer treatment, but nearly all patients relapse eventually and second-line treatment options are poor^3,4^. Except for the *KRAS* G12C mutation, the major driver genes for pancreatic cancer, *KRAS*, *TP53*, *CDKN2A*, and *SMAD4*, have not been translated into FDA-approved therapies^1^. Although recent developments have led to the clinical use of AMG 510 (sotorasib), a *KRAS*^G12C^-specific covalent inhibitor^5^, the *KRAS* G12C mutation is rare in PDAC, and no targeted therapies exist for more prevalent *KRAS* mutants, particularly G12D^6^. In 2019, following a phase III randomized clinical trial (the POLO trial), the FDA approved the PARP inhibitor olaparib for the treatment of *BRCA*-mutated pancreatic cancer; the limitation of this strategy, however, is that only a minority of PDAC patients harbor mutations in *BRCA1* (1.3%) or *BRCA2* (2.1%)^7^.

Immune checkpoint blockade (ICB) therapies, such as monoclonal antibodies against PD-1, PD-L1, or CTLA-4, prolong the survival of a subset of patients with melanoma, non-small cell lung cancer, or renal-cell cancer, among other cancer types^8–10^. However, except for the <1% of patients with microsatellite instability-high tumors^11^, clinical trials targeting immune checkpoint receptors or their cognate ligands have been ineffective in pancreatic cancer^9,12^. Dual ICB therapy using anti-CTLA-4 and anti-PD-L1 antibodies to target non-redundant pathways of T cell inactivation has also been unsuccessful^13^. Other therapies used in combination with checkpoint inhibitors, including chemotherapy, radiotherapy, targeted therapy, and vaccination, have been similarly disappointing^6,14–17^. Typically, PDAC exhibits an immunosuppressive tumor microenvironment (TME) that is characterized by prominent myeloid cell infiltration and the lack of active cytotoxic T cells^6,18^. Hence, it is critical to identify tumor-intrinsic and/or tumor-extrinsic targets that may enable the transformation of the PDAC TME from immunologically “cold” to “hot”, thereby enhancing the responsiveness to ICB therapy.

The stress hormone cortisol and synthetic glucocorticoids act via the glucocorticoid receptor (GR, encoded by *NR3C1*), a member of the nuclear receptor family that, upon ligand binding, translocates into the nucleus to regulate gene transcription^19,20^. Glucocorticoids have pleiotropic effects on the immune system^19^. Thus far, GR has been shown to act on certain cancer cells in a cell-autonomous fashion. For instance, GR can induce metastatic ability of breast cancer cells by upregulating the kinase ROR1^21^ and promote anti-androgen resistance of prostate cancer cells by substituting for the androgen receptor to activate target genes^22,23^. However, it is unknown whether GR signaling in cancer cells exerts an immunosuppressive effect on the TME, and if so, whether this can be exploited to sensitize “cold” tumors such as pancreatic cancer to ICB therapy.

In this work, we show that GR acts as a transcriptional activator of PD-L1 and a transcriptional repressor of the major histocompatibility complex class I (MHC-I) in pancreatic cancer cells, and that GR depletion or inhibition promotes the infiltration and activity of cytotoxic T cells, leading to enhanced immune surveillance and sensitization of pancreatic tumors to immune checkpoint inhibitors.

## Results

### GR activates PD-L1 expression and represses MHC-I expression in PDAC cells

**Fig. 1:**
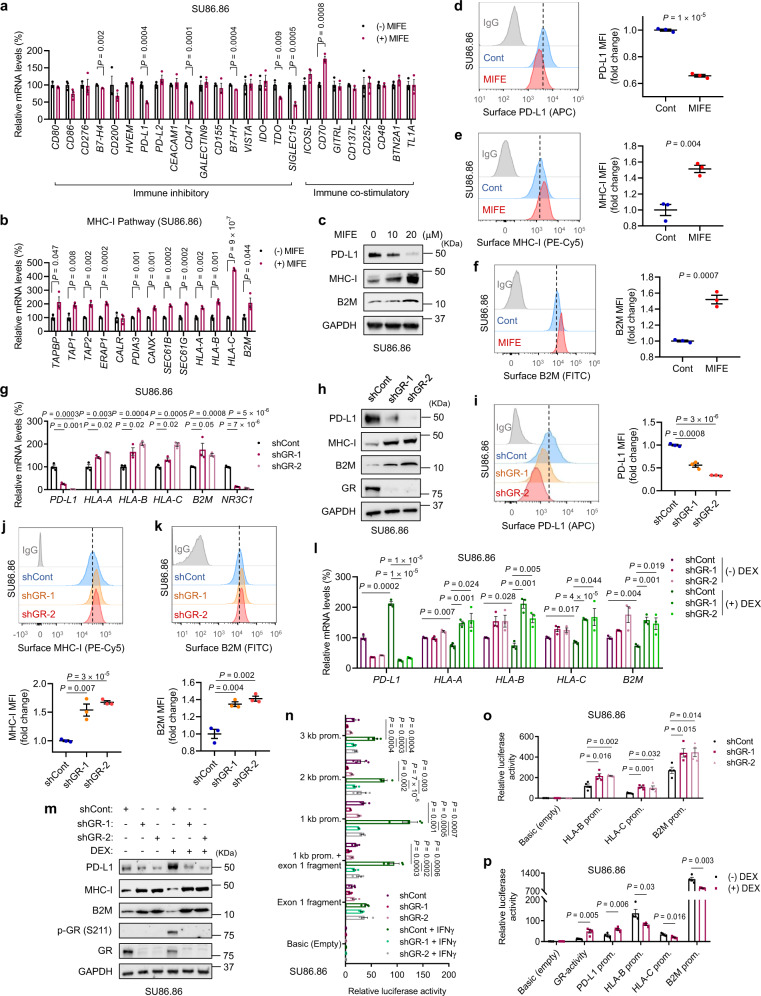
GR activates PD-L1 expression and represses MHC-I expression in human pancreatic cancer cells. **a**, **b** qPCR analysis of immune inhibitory and immune co-stimulatory genes (**a**) and genes involved in the MHC-I pathway (**b**) in SU86.86 cells with or without mifepristone (MIFE, 20 μM, 72 h) treatment. *n* = 3 technical replicates. **c** Immunoblotting of PD-L1, MHC-I, and B2M in SU86.86 cells with or without MIFE treatment with the indicated doses for 72 h. **d**–**f** Representative flow cytometry plots and quantification (by MFI: mean fluorescence intensity) of cell-surface PD-L1 (**d**), MHC-I (**e**), and B2M (**f**) in SU86.86 cells with or without MIFE (20 μM, 72 h) treatment. *n* = 3 biological replicates. **g** qPCR analysis of *PD-L1*, *HLA-A*, *HLA-B*, *HLA-C*, *B2M*, and *NR3C1* in GR-knockdown SU86.86 cells. *n* = 3 technical replicates. **h**, Immunoblotting of PD-L1, MHC-I, B2M, and GR in GR-knockdown SU86.86 cells. **i**–**k** Representative flow cytometry plots and quantification of cell-surface PD-L1 (**i**), MHC-I (**j**), and B2M (**k**) in GR-knockdown SU86.86 cells. *n* = 3 biological replicates. **l** qPCR analysis of *PD-L1*, *HLA-A*, *HLA-B*, *HLA-C*, and *B2M* in GR-knockdown SU86.86 cells, with or without dexamethasone (DEX, 100 nM, 8 h) treatment. *n* = 3 technical replicates. **m** Immunoblotting of PD-L1, MHC-I, B2M, p-GR (Ser211), and GR in GR-knockdown SU86.86 cells, with or without dexamethasone (DEX, 100 nM, 8 h) treatment. **n** Normalized luciferase activity of the reporters containing the indicated human *PD-L1* gene promoter regions in GR-knockdown SU86.86 cells, with or without IFNγ (10 ng ml^−1^, 8 h) treatment. *n* = 4 wells. **o** Normalized luciferase activity of the reporters containing the indicated human *HLA-B*, *HLA-C*, and *B2M* gene promoter regions in GR-knockdown SU86.86 cells. *n* = 4 wells. **p** Normalized luciferase activity of the reporters containing the human GR-binding elements, *PD-L1*, *HLA-B*, *HLA-C*, and *B2M* promoter regions in SU86.86 cells with or without DEX (100 nM, 8 h) treatment. *n* = 4 wells. Statistical significance in **a**, **b**, **d**–**g**, **i**–**l**, and **n**–**p** was determined by a two-tailed unpaired *t*-test. Error bars are s.e.m. Source data are provided as a Source Data file.

To determine whether GR regulates immunity-related genes in pancreatic cancer cells, we treated two human PDAC cell lines harboring the G12D hotspot mutation of *KRAS*^24,25^, SU86.86 (female) and SW1990 (male), with the clinical GR antagonist mifepristone (also known as RU486; used to treat patients with Cushing’s syndrome characterized by aberrantly high levels of glucocorticoids)^26–28^. qPCR analysis revealed that mifepristone treatment of both cell lines decreased mRNA levels of several immune checkpoint ligands^29^, including PD-L1, CD47, TDO, and SIGLEC15 (Fig. 1a and Supplementary Fig. 1a), and increased mRNA levels of several components in the MHC-I pathway, including HLA-A, HLA-B, HLA-C, B2M, SEC61B, and SEC61G (Fig. 1b and Supplementary Fig. 1b). Consistent with the effect on mRNA, PD-L1 protein was also downregulated by mifepristone treatment, as gauged by western blot analysis (Fig. 1c and Supplementary Fig. 1c) and flow cytometric analysis (Fig. 1d and Supplementary Fig. 1d). Similarly, upregulation of MHC-I and β-2-microglobulin (B2M, the common light chain of the MHC complex) proteins was observed in both SU86.86 and SW1990 cell lines treated with the GR antagonist (Fig. 1c, e, f and Supplementary Fig. 1c, e, f).

Whereas the MHC-I antigen presentation pathway is crucial for the recognition of tumor cells by CD8^+^ T cells^30^, PD-L1 is one of the key immune inhibitory ligands expressed by cancer cells^31^. Upon binding to its cognate receptor PD-1 on tumor-infiltrating cytotoxic T lymphocytes (CTLs), PD-L1 induces an inhibitory signal to dampen their tumor-killing activity^32^. To further corroborate the role of GR in regulating PD-L1 and MHC-I expression levels in pancreatic cancer cells, we knocked down GR in SU86.86 and SW1990 cell lines by two independent shRNAs, finding that silencing of GR significantly downregulated PD-L1 and upregulated MHC-I and B2M at both mRNA and protein levels (Fig. 1g–k and Supplementary Fig. 1g, h). Treatment of both PDAC cell lines with a clinical GR agonist, dexamethasone (DEX), led to upregulation of PD-L1 and downregulation of MHC-I and B2M, which could be reversed by knockdown of GR (Fig. 1l, m and Supplementary Fig. 1i, j).

GR can either activate or repress gene transcription^19^. We asked whether GR regulates the transcription of PD-L1 (encoded by *CD274*) and MHC-I genes. By using a series of luciferase reporter constructs containing previously described promoter fragments cloned from the human *CD274* gene^33^, we found that IFNγ (known to activate PD-L1 transcription)^33^ induced the activity of the promoter reporters in both SU86.86 and SW1990 cells, which was abrogated by knockdown of GR (Fig. 1n and Supplementary Fig. 1k). Moreover, silencing of GR upregulated, and dexamethasone treatment downregulated, the activity of the luciferase reporter containing the promoter of *HLA-B*, *HLA-C*, or *B2M* (Fig. 1o, p). In addition, we analyzed the promoter regions of PD-L1, HLA-A, HLA-B, HLA-C, and B2M genes and identified multiple glucocorticoid response elements (GREs). We then designed PCR amplicons for genomic regions encompassing these putative GR-binding sites (Supplementary Fig. 2a). Chromatin immunoprecipitation (ChIP)-qPCR analysis of SU86.86 cells revealed that for each of the five genes, at least one predicted binding site met the following criteria: the binding to GR was significantly induced by dexamethasone treatment, which was reversed by co-treatment with mifepristone (Supplementary Fig. 2b–f). On the other hand, treatment with the proteasome inhibitor MG132 or the lysosome inhibitor chloroquine did not affect mifepristone-mediated PD-L1 downregulation and MHC-I upregulation (Supplementary Fig. 2g), indicating that GR regulates PD-L1 and MHC-I expression independently of the proteasomal or lysosomal pathway. Collectively, these results provide evidence for the direct transcriptional regulation of PD-L1 and MHC-I genes by GR.

**Fig. 2:**
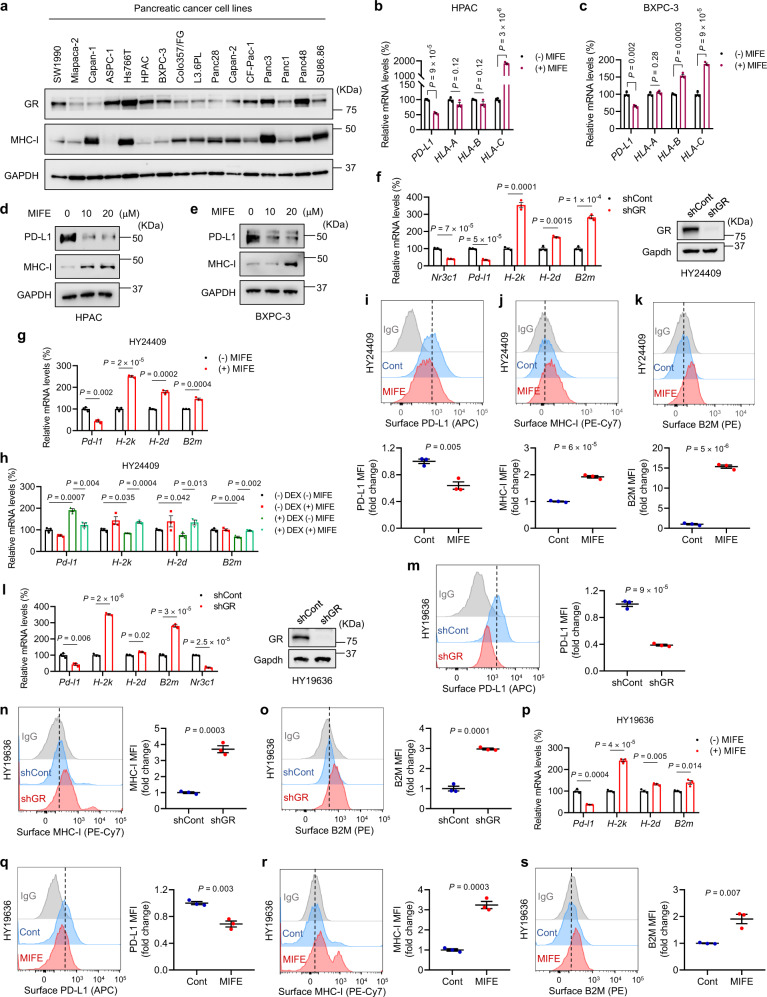
Modulation of PD-L1 and MHC-I by GR is a common regulatory mechanism in PDAC cells. **a** Immunoblotting of GR, MHC-I, and GAPDH in human PDAC cell lines. **b**, **c** qPCR analysis of *PD-L1*, *HLA-A*, *HLA-B*, and *HLA-C* in HPAC (**b**) and BXPC-3 (**c**) cells with or without MIFE (20 μM, 72 h) treatment. *n* = 3 technical replicates. **d**, **e** Immunoblotting of PD-L1, MHC-I, and GAPDH in HPAC (**d**) and BXPC-3 (**e**) cells with or without MIFE treatment with the indicated doses for 72 h. **f** Left panel: qPCR analysis of *Nr3c1*, *Pd-l1*, *H-2k*, *H-2d*, and *B2m* in GR-knockdown HY24409 cells. Right panel: immunoblotting of GR and Gapdh in GR-knockdown HY24409 cells. **g** qPCR analysis of *Pd-l1*, *H-2k*, *H-2d*, and *B2m* in HY24409 cells with or without MIFE (20 μM, 48 h) treatment. **h** qPCR analysis of *Pd-l1*, *H-2k*, *H-2d*, and *B2m* in HY24409 cells treated with DEX (100 nM, 4 h) and MIFE (100 nM, 4 h), alone or in combination. *n* = 3 technical replicates in **f**–**h**. **i**–**k** Representative flow cytometry plots and quantification of cell-surface PD-L1 (**i**), MHC-I (**j**), and B2M (**k**) in HY24409 cells with or without MIFE (20 μM, 48 h) treatment. *n* = 3 biological replicates. **l** Left panel: qPCR analysis of *Pd-l1*, *H-2k*, *H-2d*, *B2m*, and *Nr3c1* in GR-knockdown HY19636 cells. *n* = 3 technical replicates. Right panel: immunoblotting of GR and Gapdh in GR-knockdown HY19636 cells. **m**–**o** Representative flow cytometry plots and quantification of cell-surface PD-L1 (**m**), MHC-I (**n**), and B2M (**o**) in GR-knockdown HY19636 cells. *n* = 3 biological replicates. **p** qPCR analysis of *Pd-l1*, *H-2k*, *H-2d*, and *B2m* in HY19636 cells with or without MIFE (20 μM, 48 h) treatment. *n* = 3 technical replicates. **q**–**s** Representative flow cytometry plots and quantification of cell-surface PD-L1 (**q**), MHC-I (**r**), and B2M (**s**) in HY19636 cells with or without MIFE (20 μM, 48 h) treatment. *n* = 3 biological replicates. Statistical significance in **b**, **c**, and **f**–**s** was determined by a two-tailed unpaired *t*-test. Error bars are s.e.m. Source data are provided as a Source Data file.

To further determine whether modulation of PD-L1 and MHC-I by GR is a general regulatory mechanism in PDAC, we examined MHC-I and GR protein levels in 16 human PDAC lines, finding that the HPAC (female) and BXPC-3 (female) cell lines showed high GR expression and low MHC-I expression (Fig. 2a). Mifepristone treatment of HPAC and BXPC-3 cells significantly upregulated MHC-I and downregulated PD-L1 at both mRNA and protein levels (Fig. 2b–e), validating that the GR antagonist can increase MHC-I expression in PDAC cell lines with low MHC-I expression. Consistent with the results from human PDAC cell lines, knockdown of GR in a male mouse PDAC cell line HY24409 reduced PD-L1 mRNA levels and elevated mRNA levels of H-2k, H-2d, and B2m (Fig. 2f). Moreover, mifepristone treatment of HY24409 cells led to a decrease in PD-L1 mRNA levels and an increase in MHC-I and B2M mRNA levels, whereas dexamethasone treatment showed the opposite effect (Fig. 2g, h). Similarly, mifepristone treatment decreased surface PD-L1 levels and increased surface protein levels of MHC-I (H-2K^b^) and B2M (Fig. 2i–k). In addition, in a female mouse PDAC cell line HY19636, knockdown of GR (Fig. 2l–o) or mifepristone treatment (Fig. 2p–s) downregulated PD-L1 and upregulated MHC-I and B2M at mRNA and surface protein levels. Taken together, our results suggest that GR activates PD-L1 expression and represses MHC-I expression in human and mouse PDAC cells in general, regardless of sex.

### Tumor cell-specific GR depletion or pharmacologic GR inhibition promotes anti-tumor immunity in PDAC

**Fig. 3:**
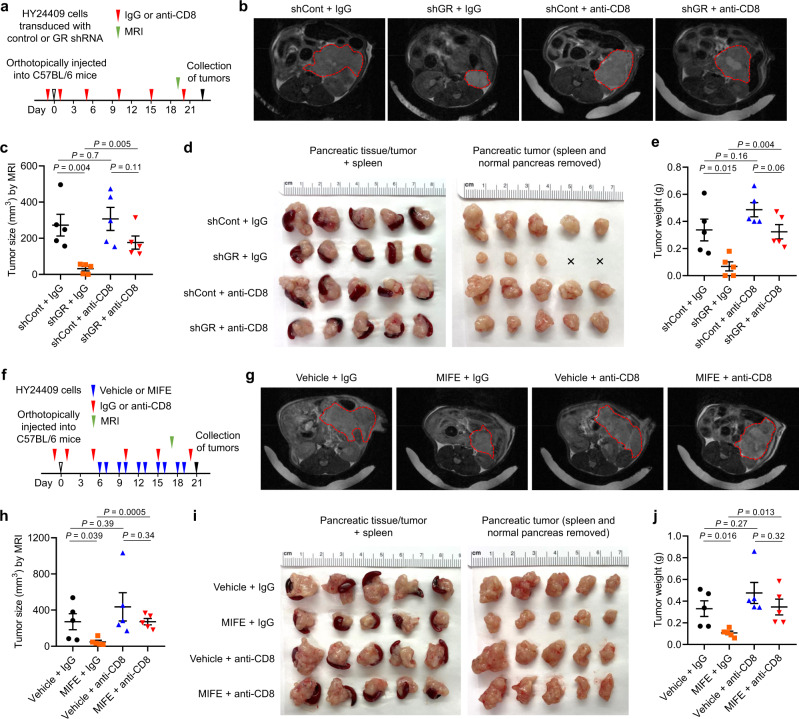
Tumor cell-specific GR depletion or pharmacologic GR inhibition suppresses pancreatic tumor growth. **a**–**e** C57BL/6 mice bearing orthotopic pancreatic tumors (implanted with 8 × 10^4^ luciferase-labeled HY24409 cells transduced with control shRNA or GR shRNA) received isotype control (IgG) or anti-CD8 antibody treatment. *n* = 5 mice. **a** Study design. MRI: magnetic resonance imaging. **b**, **c** Representative magnetic resonance images (**b**) and tumor size quantification (**c**) on day 19 after tumor cell implantation. **d**, Endpoint images of orthotopic HY24409 tumors expressing either control shRNA or GR shRNA, with or without CD8^+^ T cell depletion. **e**, Endpoint tumor weight. **f**–**j** C57BL/6 mice bearing orthotopic pancreatic tumors (implanted with 8 × 10^4^ luciferase-labeled HY24409 cells) received vehicle or mifepristone (MIFE) and isotype control (IgG) or anti-CD8 antibody treatment. *n* = 5 mice. **f** Study design. MRI: magnetic resonance imaging. **g**, **h** Representative magnetic resonance images (**g**) and tumor size quantification (**h**) on day 17 after tumor cell implantation. **i** Endpoint images of vehicle- and MIFE-treated orthotopic HY24409 tumors, with or without CD8^+^ T cell depletion. **j** Endpoint tumor weight. Statistical significance in **c**, **e**, **h**, and **j** was determined by a two-tailed unpaired *t*-test. Error bars are s.e.m. Source data are provided as a Source Data file.

To determine the functional role of GR in pancreatic cancer cells, we implanted either NSG (non-obese diabetic; severe combined immunodeficiency; interleukin-2 receptor gamma chain null) mice or immunocompetent C57BL/6 mice with two mouse PDAC cell lines (HY24409 and HY24160), both of which were derived from male KPC (p48-Cre;*Kras*^LSL-G12D/+^;*Trp53*^loxP/+^) mice^34,35^ that were backcrossed to the C57BL/6 background (the purity of the C57BL/6 background of the mice used for KPC cell line generation was approximately 98% based on SNP analysis). We then treated the mice with mifepristone via oral gavage (60 mg kg^−1^, twice every 3 days). In NSG mice, neither mifepristone treatment (Supplementary Fig. 3a–j) nor shRNA-mediated GR knockdown (Supplementary Fig. 3k–n) affected the growth of tumors formed by KPC cell lines. In contrast, in C57BL/6 mice, either GR knockdown in HY24409 cells (Fig. 3a–e) or systemic mifepristone treatment (Fig. 3f–j) led to substantial reductions in orthotopic pancreatic tumor volume (gauged by magnetic resonance imaging) and weight, without significant loss of body weight (Supplementary Fig. 3o, p). At the endpoint, we observed 80% and 67.7% reductions in tumor weight by GR knockdown (Fig. 3d, e) and mifepristone treatment (Fig. 3i, j), respectively. Flow cytometric analysis of HY24409 cells collected from unsynchronized conditions and at different time points after release from double thymidine block (which arrests most cells at G1/S boundary, prior to DNA replication) showed that neither GR knockdown (Supplementary Fig. 3q, r) nor mifepristone treatment (Supplementary Fig. 3s, t) altered the cell cycle profile. Altogether, these data indicate that the TME, mostly likely the immune components, are involved in the observed tumor growth inhibition.

**Fig. 4:**
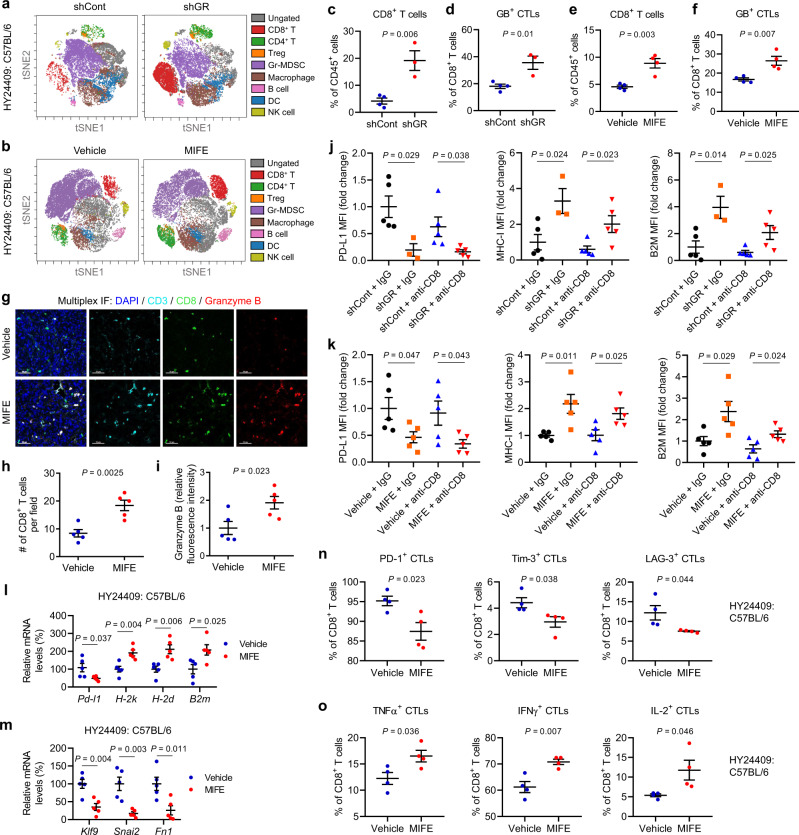
Tumor cell-specific GR depletion or pharmacologic GR inhibition promotes anti-tumor immunity in PDAC. **a**, **b** CyTOF-based immune profiling of orthotopic HY24409 tumors expressing control shRNA or GR shRNA (**a**), and of vehicle- and MIFE-treated orthotopic HY24409 tumors (**b**). Representative viSNE plots were colored by immune cell populations. **c**, **d** Quantification of CD8^+^ T cells (**c**) and granzyme B (GB)^+^ CTLs (**d**) in orthotopic HY24409 tumors expressing control shRNA (*n* = 4 mice) or GR shRNA (*n* = 3 mice). **e**, **f** Quantification of CD8^+^ T cells (**e**) and granzyme B (GB)^+^ CTLs (**f**) in vehicle- and MIFE-treated orthotopic HY24409 tumors. *n* = 4 mice. **g**–**i** Multiplex immunofluorescent staining of CD3, CD8, and granzyme B in vehicle- and MIFE-treated orthotopic HY24409 tumors (**g**), and quantification of CD8 (**h**) and granzyme B (**i**) signals. Scale bars, 50 μm. *n* = 5 mice. **j** Flow cytometric analysis of cell-surface PD-L1 (left), MHC-I (H-2K^b^) (middle), and B2M (right) levels in cancer cells sorted from orthotopic HY24409 tumors expressing control shRNA or GR shRNA, with or without CD8^+^ T cell depletion. *n* = 5, 3, 5, and 5 mice from left to right. **k** Flow cytometric analysis of cell-surface PD-L1 (left), MHC-I (H-2K^b^) (middle), and B2M (right) levels in cancer cells sorted from vehicle- and MIFE-treated orthotopic HY24409 tumors, with or without CD8^+^ T cell depletion. *n* = 5 mice. **l** qPCR analysis of *Pd-l1*, *H-2k*, *H-2d*, and *B2m* in vehicle- and MIFE-treated orthotopic HY24409 tumors. *n* = 5 mice. **m** qPCR analysis of known GR-activated target genes in vehicle- and MIFE-treated orthotopic HY24409 tumors. *n* = 5 mice. **n** Quantification of PD-1^+^, Tim-3^+^, and LAG-3^+^ CTLs in vehicle- and MIFE-treated orthotopic HY24409 tumors. *n* = 4 mice. **o** Quantification of TNFα, IFNγ, and IL-2 expression in intratumoral CD8^+^ T cells from orthotopic HY24409 tumors, after ex vivo phorbol myristate acetate (PMA)/ionomycin stimulation. *n* = 4 mice. Statistical significance in **c**–**f** and **h**–**o** was determined by a two-tailed unpaired *t*-test. Error bars are s.e.m. Source data are provided as a Source Data file.

To assess the effect of tumor cell-specific GR depletion or pharmacologic GR inhibition on the tumor immune microenvironment, we used time-of-flight mass cytometry (CyTOF) for high-dimensional analysis of tumor-associated immune cells at the single-cell level^36,37^ (Fig. 4a, b). This analysis revealed that in pancreatic tumors generated by HY24409 cells, either GR knockdown or mifepristone treatment significantly increased the abundance and activity of CTLs, as gauged by CD8 (a marker of CTLs) and granzyme B (a marker of CTL activity), respectively (Fig. 4a–f). Specifically, knockdown of GR increased the percentages of tumor-infiltrating CD8^+^ cells and granzyme B^+^ CTLs by 4.6-fold and 2-fold (Fig. 4c, d), while treatment with mifepristone increased the percentages of tumor-infiltrating CD8^+^ cells and granzyme B^+^ CTLs by 1.9-fold and 1.6-fold (Fig. 4e, f). On the other hand, we found no significant change in the abundance of CD4^+^ T cells, regulatory T cells, natural killer cells, B cells, dendritic cells, macrophages, and myeloid-derived suppressor cells (Supplementary Fig. 4a, b). In parallel, we performed multiplex immunofluorescent staining of CD3 (a marker of T cells), CD8, and granzyme B, finding a substantial enrichment of these three markers in pancreatic tumors from mifepristone-treated C57BL/6 mice (Fig. 4g–i), further confirming the increase in tumor infiltration by active CTLs. Notably, antibody-mediated depletion of CD8^+^ T cells (Supplementary Fig. 4c) abolished the tumor growth inhibition caused by GR knockdown (Fig. 3b–e) or mifepristone treatment (Fig. 3g–j), suggesting that CTLs mediate the observed anti-tumor effect of GR depletion or inhibition.

Consistent with the observed anti-tumor effect of GR knockdown or inhibition in immunocompetent mice, either GR-depleted or mifepristone-treated pancreatic tumors showed lower cell-surface PD-L1 levels as well as higher cell-surface MHC-I (H-2K^b^) and B2M levels, as gauged by flow cytometric analysis (Fig. 4j, k and Supplementary Fig. 4d, e). Moreover, mRNA levels of PD-L1 and known GR-activated target genes (*Klf9*, *Snai2*, and *Fn1*)^21^ were downregulated in mifepristone-treated tumors (Fig. 4l, m), whereas mRNA levels of MHC-I components (H-2k, H-2d, and B2m) were upregulated (Fig. 4l). Next, we profiled chemokines secreted by HY24409 cells. Several chemokines, including CXCL16, KC, LIX, and MIP-2, were present at high levels in the conditioned medium of HY24409 cells; however, we did not find chemokines that showed a change after mifepristone treatment (Supplementary Fig. 4f). We also examined PD-1, Tim-3, and LAG-3 by flow cytometry, finding a significant reduction in surface levels of these T cell exhaustion markers in mifepristone-treated HY24409 pancreatic tumors (Fig. 4n). Furthermore, we performed flow cytometric analysis of intracellular TNFα, IFNγ, and IL-2 on tumor-associated CD8^+^ cells after ex vivo stimulation with phorbol myristate acetate (PMA) and ionomycin, and we found that mifepristone treatment significantly increased expression levels of these three cytokines (Fig. 4o). Taken together, our data suggest that GR inhibition elevates the number and activity of tumor-infiltrating CD8^+^ T cells.

Similar to the results from the HY24409 model, in orthotopic pancreatic tumors formed by the HY24160 cell line, treatment with mifepristone also inhibited tumor growth (without affecting body weight) in a CTL-dependent manner (Supplementary Fig. 5a–e), promoted the infiltration and activity of cytotoxic T cells (Supplementary Fig. 5f–k), downregulated expression levels of PD-L1 and known GR-activated genes (Supplementary Fig. 5l–o), and upregulated MHC-I expression levels (Supplementary Fig. 5l–n). Collectively, these data suggest that GR promotes pancreatic cancer immune evasion.

**Fig. 5:**
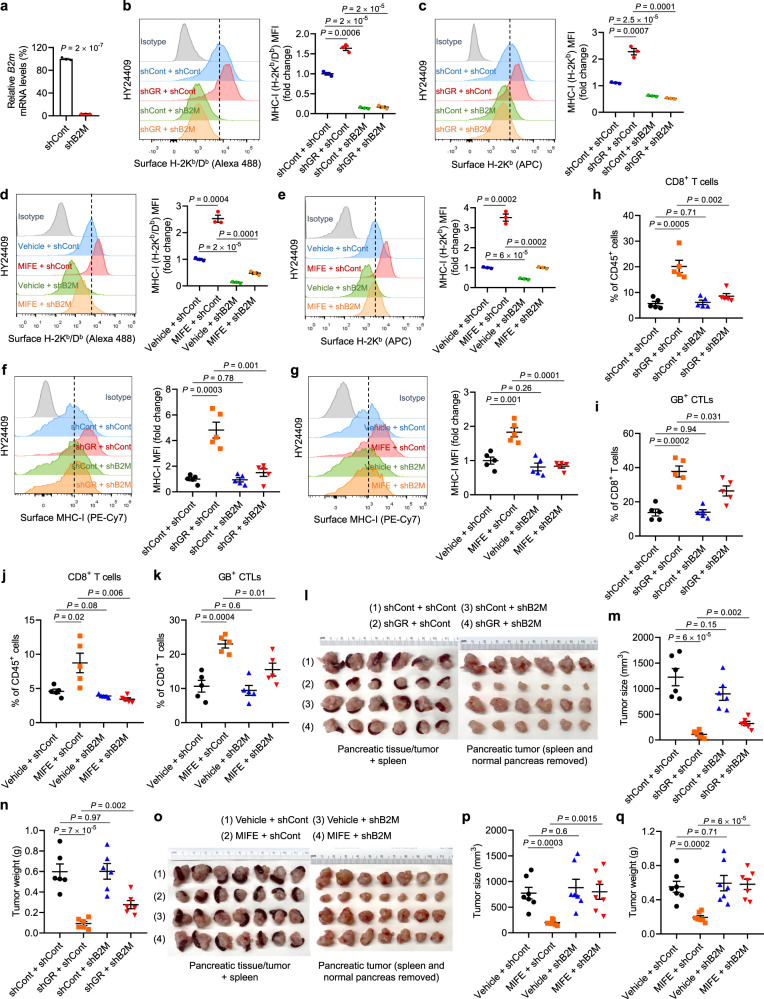
GR-mediated regulation of MHC-I is required for the anti-tumor effect of GR depletion or inhibition. **a** qPCR analysis of *B2m* in HY24409 cells transduced with control shRNA or B2M shRNA. *n* = 3 technical replicates. **b**, **c** Representative flow cytometry plots and quantification (by MFI: mean fluorescence intensity) of cell-surface H-2K^b^/D^b^ (**b**) and H-2K^b^ (**c**) in HY24409 cells transduced with GR shRNA and B2M shRNA, alone or in combination. Cells were treated with IFNγ (10 ng ml^−1^, overnight). *n* = 3 biological replicates. **d**, **e** Representative flow cytometry plots and quantification of cell-surface H-2K^b^/D^b^ (**d**) and H-2K^b^ (**e**) in HY24409 cells transduced with control shRNA or B2M shRNA, with or without mifepristone (MIFE) treatment (20 μM, 48 h). Cells were treated with IFNγ (10 ng ml^−1^, overnight). *n* = 3 biological replicates. **f**, **g** Representative flow cytometry plots and quantification of cell-surface MHC-I (H-2K^b^) in orthotopic HY24409 tumors of the indicated groups. *n* = 5 mice. **h**–**k** Quantification of CD8^+^ T cells (**h**, **j**) and granzyme B (GB)^+^ CTLs (**i**, **k**) in orthotopic HY24409 tumors of the indicated groups. *n* = 5 mice. **l**, **m** Endpoint (22 days after inoculation) tumor images (**l**), tumor size (**m**), and tumor weight (**n**) of C57BL/6 mice bearing orthotopic pancreatic tumors. Mice were implanted with 8 × 10^4^ luciferase-labeled HY24409 cells transduced with GR shRNA and B2M shRNA, alone or in combination. *n* = 6 mice. **o**–**q** Endpoint (22 days after inoculation) tumor images (**o**), tumor size (**p**), and tumor weight (**q**) of C57BL/6 mice bearing orthotopic pancreatic tumors. Mice were implanted with 8 × 10^4^ luciferase-labeled HY24409 cells transduced with control shRNA or B2M shRNA, and were treated with vehicle or mifepristone (MIFE) twice every 3 days. *n* = 7 mice. Statistical significance in **a**–**k**, **m**, **n**, **p**, and **q** was determined by a two-tailed unpaired *t*-test. Error bars are s.e.m. Source data are provided as a Source Data file.

To determine whether GR-mediated regulation of MHC-I is required for the observed anti-tumor effect of GR depletion or inhibition, we knocked down B2M, an essential structural component of the MHC-I complex, in HY24409 cells (Fig. 5a), which abrogated the upregulation of surface MHC-I induced by GR depletion or inhibition (Fig. 5b–e). In GR-knockdown or mifepristone-treated HY24409 pancreatic tumors, knockdown of B2M depleted surface MHC-I in vivo (Fig. 5f, g), decreased the percentages of tumor-infiltrating CD8^+^ T cells and granzyme B^+^ CTLs (Fig. 5h–k), and rescued tumor growth (Fig. 5l–q). Taken together with our CD8^+^ T cell depletion data, these results demonstrate that increased surface expression of MHC-I on pancreatic tumor cells upon GR knockdown or inhibition is a prerequisite for increased CD8^+^ T cell infiltration and tumor suppression.

### Tumor cell-specific GR depletion or pharmacologic GR inhibition sensitizes PDAC to immunotherapy

**Fig. 6:**
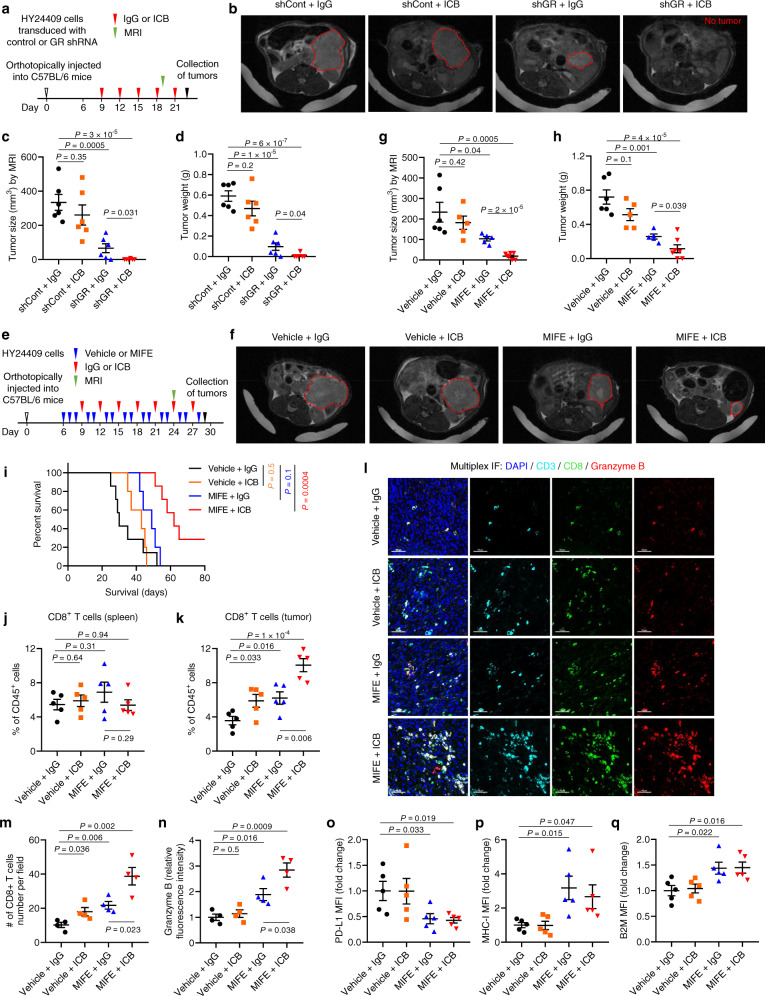
Tumor cell-specific GR depletion or pharmacologic GR inhibition sensitizes PDAC to immunotherapy in male mice. **a**–**d** C57BL/6 mice bearing orthotopic pancreatic tumors (implanted with 8 × 10^4^ luciferase-labeled HY24409 cells transduced with control shRNA or GR shRNA) were treated with isotype control (IgG) or dual ICB (anti-PD1 and anti-CTLA-4 monoclonal antibodies). *n* = 6 mice. **a** Study design. MRI: magnetic resonance imaging. **b**, **c** Representative magnetic resonance images (**b**) and tumor size quantification (**c**) on day 19 after tumor cell implantation. **d** Endpoint tumor weight. **e**–**h** C57BL/6 mice bearing orthotopic pancreatic tumors (implanted with 4 × 10^4^ luciferase-labeled HY24409 cells) were treated with mifepristone (MIFE) and dual ICB, alone or in combination. *n* = 6, 5, 5, and 7 mice from left to right. **e** Study design. MRI: magnetic resonance imaging. **f**, **g** Representative magnetic resonance images (**f**) and tumor size quantification (**g**) on day 24 after tumor cell implantation. **h** Endpoint tumor weight. **i** Overall survival curves of HY24409 tumor-bearing C57BL/6 mice treated with mifepristone (MIFE) and dual ICB, alone or in combination. Statistical significance was determined by the log-rank test. *n* = 7, 5, 5, and 7 mice from left to right. **j**–**q** Flow cytometric analysis and multiplex immunofluorescent staining of tissues (orthotopic HY24409 tumors or spleens) from C57BL/6 mice treated with mifepristone (MIFE) and dual ICB, alone or in combination. **j**, **k** Quantification of CD8^+^ T cells in the spleens (**j**) and pancreatic tumors (**k**) by flow cytometry. *n* = 5 mice. **l**–**n** Multiplex immunofluorescent staining of CD3, CD8, and granzyme B in the tumors (**l**), and the quantification of CD8 (**m**) and granzyme B (**n**) signals. Scale bars, 50 μm. *n* = 4 mice. **o**–**q** Flow cytometric analysis of cell-surface PD-L1 (**o**), MHC-I (H-2K^b^) (**p**), and B2M (**q**) in tumor cells (gated by ZombieDye^–^CD45^–^luciferase^+^). MFI: mean fluorescence intensity. *n* = 5 mice. Statistical significance in **c**, **d**, **g**, **h**, **j**, **k**, and **m**–**q** was determined by a two-tailed unpaired *t*-test. Error bars are s.e.m. Source data are provided as a Source Data file.

Pancreatic cancer is highly resistant to ICB therapy; to date, even targeting multiple immune checkpoints has failed in clinical trials^18^. Similarly, female mice with orthotopic implantation of the female KPC line HY15549 do not respond to ICB by anti-CTLA-4 and anti-PD-1 treatment, even when used in combination^35^. To corroborate this result, we treated male C57BL/6 mice bearing orthotopic pancreatic tumors formed by the male KPC line HY24409 with dual ICB drugs (anti-CTLA-4 and anti-PD-l antibodies), finding no significant effect on tumor size or weight (Fig. 6a–d). Strikingly, shRNA-mediated knockdown of GR in HY24409 cells rendered otherwise ICB-resistant orthotopic pancreatic tumors highly sensitive to dual ICB treatment (Fig. 6a–d and Supplementary Fig. 6a), without causing body weight loss (Supplementary Fig. 6b), which underscores a tumor cell-specific role of GR in regulating immunotherapeutic response.

Next, we assessed the translatability of our results, finding that the combination treatment with mifepristone and dual ICB achieved a greater anti-tumor effect than mifepristone as a single agent (Fig. 6e–h and Supplementary Fig. 6c, d). Importantly, compared with the vehicle + IgG control, the combination therapy markedly prolonged survival in mice bearing orthotopic pancreatic tumors (log-rank *P* = 0.0004, hazard ratio = 0.19, median survival: 30 days vs. 62 days, Fig. 6i); in contrast, either dual ICB therapy (*P* = 0.5) or mifepristone treatment alone (*P* = 0.1) did not lead to a significant improvement in survival (Fig. 6i). Thus, treatment with the clinical GR antagonist mifepristone can sensitize ICB-refractory PDAC to anti-CTLA-4 and anti-PD-l antibodies, resulting in not only substantial tumor growth inhibition but also significant survival benefit.

Similar to the experiments described above (Fig. 4g–i and Supplementary Fig. 5i–k), we reproducibly observed an increase in tumor infiltration by CD8^+^ T cells and granzyme B^+^ CTLs, but not in splenic CD8^+^ T cells, upon systemic mifepristone treatment, based on flow cytometric analysis and multiplex immunofluorescent staining (Fig. 6j–n). Dual ICB treatment also increased tumor-infiltrating CD8^+^ T cells, but did not increase the activity of CTLs, as gauged by granzyme B (Fig. 6k–n). Notably, the combination treatment with mifepristone and dual ICB further augmented the abundance of tumor-infiltrating CD8^+^ T cells and granzyme B^+^ CTLs, compared with mifepristone treatment alone (Fig. 6k–n). Relative to the control group, mifepristone-treated pancreatic tumors showed a decrease in cell-surface PD-L1 (Fig. 6o) as well as an increase in cell-surface MHC-I (H-2K^b^; Fig. 6p) and B2M (Fig. 6q), either with or without co-treatment with dual ICB.

**Fig. 7:**
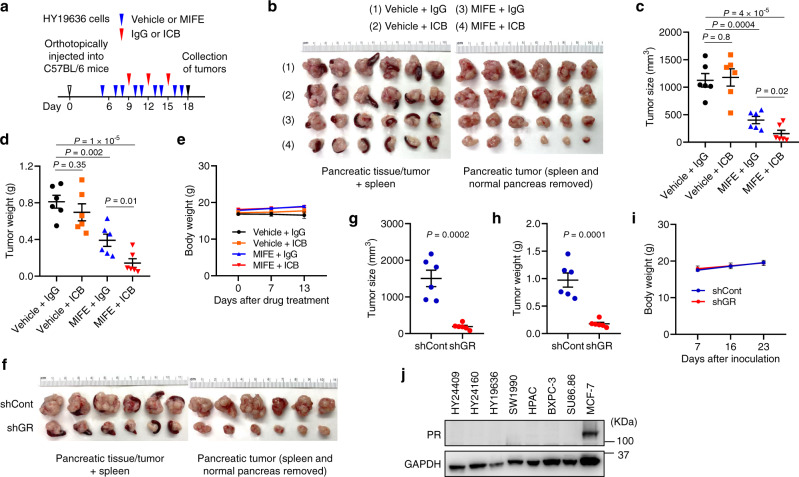
Depletion or inhibition of GR suppresses pancreatic tumor growth and renders sensitivity to immunotherapy in female mice. **a**–**e** C57BL/6 mice bearing orthotopic pancreatic tumors (implanted with 4 × 10^4^ RFP-labeled HY19636 cells) were treated with mifepristone (MIFE) and dual ICB (anti-PD1 and anti-CTLA-4 monoclonal antibodies), alone or in combination. *n* = 6 mice. **a** Study design. **b** Endpoint tumor images. **c** Endpoint tumor size. **d** Endpoint tumor weight. **e** Body weight. **f**–**i** C57BL/6 mice were orthotopically implanted with 8 × 10^4^ RFP-labeled HY19636 cells transduced with control shRNA or GR shRNA. *n* = 6 mice. **f** Endpoint tumor images. **g** Endpoint tumor size. **h** Endpoint tumor weight. **i** Body weight. **j** Immunoblotting of PR and GAPDH in the indicated mouse and human pancreatic cancer cell lines. The MCF-7 cell line was used as a positive control. Statistical significance in **c**, **d**, **g**, and **h** was determined by a two-tailed unpaired *t*-test. Error bars are s.e.m. Source data are provided as a Source Data file.

Furthermore, in female C57BL/6 mice bearing orthotopic tumors formed by the female KPC cell line, HY19636, treatment with mifepristone markedly inhibited tumor growth and rendered sensitivity to dual ICB, without reducing body weight (Fig. 7a–e). In addition, knockdown of GR in HY19636 cells substantially reduced orthotopic pancreatic tumor size and weight, without altering the body weight of female C57BL/6 hosts (Fig. 7f–i), which underscores the importance of tumor cell-specific GR. Altogether, our findings hold true in both males and females. It should be noted that mifepristone is an antagonist of both GR and the progesterone receptor (PR)^26,27^. However, the mouse and human PDAC cell lines used in this study showed no detectable PR expression (Fig. 7j; an ER^+^PR^+^ breast cancer cell line, MCF-7, was used as a positive control). Thus, the mifepristone effects in our study can be attributed to GR.

### GR correlates with PD-L1 expression, low MHC-I expression, and poor survival in PDAC

**Fig. 8:**
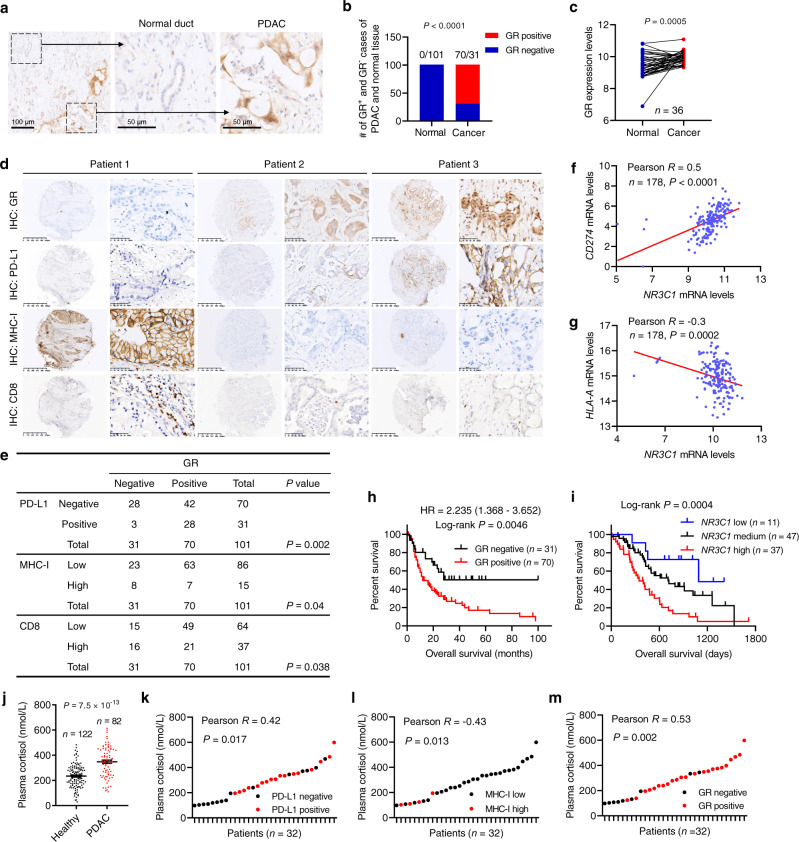
GR correlates with PD-L1 expression, low MHC-I expression, and poor survival in human PDAC. **a** Representative immunohistochemical (IHC) staining of GR in the normal pancreatic duct and PDAC. Scale bars, 100 μm (left), 50 μm (middle), and 50 μm (right). **b** Quantification of GR-positive and GR-negative cases of PDAC and adjacent normal pancreatic tissue. *n* = 101 patients. Statistical significance was determined by a two-tailed Fisher’s exact test. **c** GR (encoded by *NR3C1*) mRNA levels in paired normal pancreatic tissue and PDAC based on the GSE15471 dataset. *n* = 36 patients. Statistical significance was determined by a two-tailed paired *t*-test. **d**, **e** Representative IHC staining (**d**) and statistical analysis (**e**) of the correlation of GR protein levels with PD-L1, MHC-I, and CD8 proteins levels in patients with PDAC. *n* = 101 patients. **f**, **g** Correlation of *NR3C1* (encoding GR) mRNA levels with *CD274* (encoding PD-L1; **f**) and *HLA-A* (**g**) mRNA levels in PDAC based on TCGA data. *n* = 178 patients. **h** Kaplan–Meier curves of overall survival of pancreatic cancer patients stratified by GR protein levels based on IHC. *n* = 101 patients. **i** Kaplan–Meier curves of overall survival of pancreatic patients stratified by GR (encoded by *NR3C1*) mRNA levels. Data were obtained from the ICGC (International Cancer Genome Consortium). *n* = 95 patients. Statistical significance was determined by the log-rank test in **h** and **i**. **j** Plasma cortisol levels in healthy volunteers (*n* = 122) and PDAC patients (*n* = 82). Statistical significance was determined by a two-tailed unpaired *t*-test. Error bars are s.e.m. **k**–**m** Correlation of plasma cortisol levels with PD-L1 (**k**), MHC-I (**l**), or GR (**m**) protein levels in pancreatic tumors. *n* = 32 patients. Statistical significance was determined by a two-tailed Pearson correlation test in **e**–**g** and **k**–**m**. Source data are provided as a Source Data file.

To assess the relevance of GR in human pancreatic cancer, we constructed pancreatic tissue microarrays (TMAs) from 101 patients with PDAC and performed immunohistochemical (IHC) staining of GR. Notably, 70 of 101 PDAC tumors showed positive GR staining, whereas none of the adjacent normal pancreatic duct tissues were GR-positive (Fig. 8a, b). Consistently, based on the gene expression data from paired samples (GSE15471)^38^, mRNA levels of GR (encoded by *NR3C1*) were upregulated in PDAC relative to paired normal pancreatic tissue (Fig. 8c).

We immunostained the TMAs for PD-L1, MHC-I, and CD8, finding that 28 of 31 (90.3%) GR-negative PDAC tumors were also negative for PD-L1, whereas 63 of 70 (90%) GR-positive PDAC tumors had low levels of MHC-I (Fig. 8d, e). Moreover, tumoral GR protein correlated with low levels of tumor-infiltrating CD8^+^ cells (Fig. 8d, e). Similarly, analysis of the gene expression data from The Cancer Genome Atlas (TCGA) revealed a positive correlation of *NR3C1* mRNA levels with *CD274* (encoding PD-L1) mRNA levels and a moderate inverse correlation of *NR3C1* mRNA levels with *HLA-A* mRNA levels in pancreatic cancer (Fig. 8f, g). The TMA data analysis indicated that patients with GR-positive PDAC had much shorter overall survival than patients with GR-negative PDAC (Fig. 8h). Similarly, based on analysis of the International Cancer Genome Consortium (ICGC) data, higher expression of *NR3C1* correlated with worse survival in pancreatic cancer patients (Fig. 8i). Taken together, these results indicate that GR expression correlates with PD-L1 expression, low MHC-I expression, low CD8^+^ T cell infiltration, and poor survival in patients with pancreatic cancer. Intriguingly, plasma levels of cortisol, the natural agonist of GR in humans, were significantly elevated in patients with PDAC compared with healthy volunteers (Fig. 8j). In PDAC patients, plasma cortisol levels positively correlated with tumoral PD-L1 protein expression and inversely correlated with tumoral MHC-I protein expression (Fig. 8k, l). Interestingly, we observed a significant positive correlation between plasma cortisol levels and GR proteins levels in pancreatic tumors (Fig. 8m); whether GR regulates its own expression in PDAC warrants future investigation. Collectively, our data support a link between activation of GR signaling and pancreatic cancer immune evasion.

## Discussion

Synthetic glucocorticoids, which mimic the effects of cortisol, are commonly used to manage symptoms, such as fatigue, dyspnea, and decreased appetite, in cancer patients^39–41^. Despite their effectiveness in alleviating such symptoms, concern has recently been expressed that use of corticosteroids may decrease the efficacy of immunotherapy^42^. Glucocorticoids and GR signaling are long thought to suppress immunity by acting on immune cells^19^, and this dogma has been extended to anti-tumor immunity recently. For example, psychological stress-induced glucocorticoid surges have been reported to induce TSC22D3 in dendritic cells, which blocks type-I IFN responses in dendritic cells and IFNγ^+^ T-cell activation^43^. Moreover, GR signaling in CD8^+^ T cells has been shown to transactivate immune checkpoint receptor expression^44^. In parallel, tumor cell-autonomous roles for GR have been found in breast cancer and prostate cancer^21–23^. In this study, we discovered a previously undescribed role for tumoral GR in upregulating PD-L1 and downregulating MHC-I in pancreatic cancer cells, which in turn exerts an immunosuppressive effect on the TME, resulting in immune evasion and immunotherapy resistance (Supplementary Fig. 7). Our findings are highly relevant to human PDAC, because cortisol levels in the blood and GR expression in tumor cells are elevated in patients with pancreatic cancer, and because in PDAC tumors, GR expression correlates with PD-L1 expression and inversely correlates with MHC-I expression and overall survival.

Either serum or local microenvironment can serve as a source of glucocorticoids. Normally, glucocorticoid production occurs in the adrenal cortex, and adrenal glucocorticoid biosynthesis is induced upon activation of the hypothalamic–pituitary–adrenal axis (HPA axis). Glucocorticoids released into the blood circulation exert systemic effects by binding to GR that is present in cells throughout the body^19^. Psychological stress, which is often elevated in cancer patients, is known to activate the HPA axis and increase blood glucocorticoid levels to activate GR signaling. In addition, various cell types in the TME, such as cancer cells^45^, T cells^46^, and macrophages^44^, have been reported to produce glucocorticoids.

With increasing incidences and minimal changes in mortality rates, pancreatic cancer is projected to become the second leading cause of cancer-related death by 2030^47^. Thus, while the progress in surgery, radiotherapy, chemotherapy, and targeted therapy has expanded on the limited treatment options for PDAC patients, breakthrough strategies are needed to make substantial improvements in survival. Here we found that tumor cell-specific depletion of GR, as well as systemic treatment with the clinical GR antagonist mifepristone, overcame immune evasion and sensitized PDAC tumors to dual ICB in mouse models, leading to tumor growth inhibition and prolonged survival. These findings provide a rationale to pursue clinical testing of the combination treatment of GR-positive pancreatic cancer with the GR antagonist and immunotherapy. It should be noted that in our study, knockdown of GR exhibited a greater anti-tumor effect than mifepristone treatment, which could be because mifepristone treatment was initiated 6 days after tumor cell implantation (when the tumor size reached ~50 mm^3^). Alternatively, we speculate that GR might have transcription-independent functions, which would be abolished by depletion of GR but not by the GR antagonist.

Future studies should address the following directions: first, our study is focused on pancreatic cancer; whether tumoral GR also regulates PD-L1 and MHC-I expression levels and immunotherapeutic response in other cancer types warrants future investigation. Second, although mifepristone plus dual ICB therapy did improve the survival of pancreatic tumor-bearing mice substantially, the dose and schedule may need to be further optimized to improve efficacy and/or reduce possible toxicity. Third, in our animal studies and preclinical drug testing, we used orthotopic pancreatic tumor models generated from three KPC lines. While this approach is well accepted in the field for studying immune evasion and immunotherapy resistance in PDAC^35^, whether it fully recapitulates the therapy response of human pancreatic tumors is not clear. Thus, a phase I clinical trial would be an appropriate next step. Fourth, the correlation analyses in this study were performed on patients that received chemotherapy but not immunotherapy. It should be noted that therapeutic intervention with drugs or irradiation has been shown to induce anti-cancer immune responses, and that immune cells, such as dendritic cells and cytotoxic T lymphocytes, play an important role in chemotherapy-induced immunogenic cell death^43,48–51^. Given that our work reveals GR signaling in cancer cells as a tumor-intrinsic mechanism of immune escape, further studies could help determine whether targeting GR sensitizes pancreatic cancer to chemotherapies through immune regulation. Since mifepristone is a clinical GR antagonist with well-established safety profiles, we envision timely clinical testing of mifepristone plus ICB therapy (or chemotherapy) in patients with pancreatic cancer.

## Methods

### Mouse experiments

All animal studies were performed in accordance with a protocol (protocol number: 00001012-RN02; PI: Li Ma) approved by the Institutional Animal Care and Use Committee (IACUC) of MD Anderson Cancer Center. Animals were housed at 70 °F–74 °F (set point: 72 °F) with 40–55% humidity (set point: 45%). The light cycle of animal rooms is 12 h of light and 12 of dark. Orthotopic injection of PDAC cells was performed as described previously^35^. Male and female C57BL/6 mice were from MD Anderson’s internal supply or the Jackson Laboratory. We performed the surgery when the mice were 6–8 weeks old. After the upper abdomen was shaved and the skin was disinfected, mice were anesthetized with isoflurane. A small incision was made on the upper left abdomen to expose the pancreas and the spleen. Approximately 4–8 × 10^4^ HY24409 (male), HY24160 (male), or HY19636 (female) cells, with > 95% viability in trypan blue exclusion assays, were suspended in a mixture of 10 μl phosphate-buffered saline (PBS) and 10 μl Matrigel (VWR, 47743-720) and were then injected into a region of the pancreas just beneath the spleen. A 30G1/2 needle (BD, 305106) and a 100-μl syringe (Hamilton, 80601) were used to inject the cell suspension. A successful subcapsular intrapancreatic injection of tumor cells was confirmed by the appearance of a fluid bleb without leakage. After the injection, the peritoneum was closed with absorbable sutures (Oasis, MV-J397-V) and the skin was closed using the BD AutoClip Wound Closing System (BD, 427630). We started drug treatment after confirming tumor formation by magnetic resonance imaging (MRI), and mice were randomly assigned to different treatment groups. To assess the importance of immune regulation, we used male NSG (non-obese diabetic; severe combined immunodeficiency; interleukin-2 receptor gamma chain null) mice from MD Anderson’s internal supply. Six-week-old NSG mice received subcutaneous injection of ~4–10 × 10^4^ HY24409 or HY24160 cells.

For in vivo CD8^+^ T cell depletion, mice received intraperitoneal injection of anti-mouse CD8α antibody (200 μg, Bio X Cell, BE0061, clone 2.43; RRID: AB_1125541) or rat IgG2b isotype control (200 μg, Bio X Cell, BE0090, clone LTF-2; RRID: AB_1107780) at the indicated times. Depletion was confirmed by flow cytometric analysis of dissociated tumor samples or blood samples with antibodies targeting non-competing CD8 epitopes. For immune checkpoint blockade experiments, mice received intraperitoneal injection of anti-mouse PD-1 antibody (100 μg, Bio X Cell, BE0146, clone RMP1-14; RRID: AB_10949053) and anti-mouse CTLA-4 antibody (100 μg, Bio X Cell, BE0032, clone UC10-4F10-11; RRID: AB_1107598), or rat IgG2a isotype control (100 μg, Bio X Cell, BE0089, clone 2A3; RRID: AB_1107769) and control hamster IgG (100 μg, Bio X Cell, BE0091; RRID: AB_1107773) at the indicated times. For GR antagonist treatment, mifepristone (Selleckchem, S2606) was dissolved in vehicle solvent containing 5% dimethylacetamide (Sigma-Aldrich, D137510) and 95% olive oil (Sigma-Aldrich, O1514). The dose was 60 mg kg^−1^ by oral gavage, which was calculated based on a phase 2 clinical trial (ClinicalTrials.gov, identifier: NCT02642939) and previously described dose conversion between animals and humans^52^. Mifepristone was administered on a schedule of twice every 3 days.

All animal experiments were performed in a specific pathogen-free facility of MD Anderson Cancer Center. Orthotopic pancreatic tumor size was determined by MRI. Subcutaneous tumor size was measured with a caliper. No tumors exceeded the IACUC-defined maximum diameter of 2 cm. Sample sizes were determined according to our preliminary experiments. Blinding was not performed as the investigator needed to know the treatment groups. Animal body weight was measured throughout the study. Tumor weight was measured at the study endpoint after the mice were euthanized.

### Multiplex immunofluorescent staining of mouse tumors

After euthanasia, mouse tissues were fixed in 10% neutral-buffered formalin (Sigma-Aldrich, HT501128) overnight, washed with PBS, transferred to 70% ethanol, embedded in paraffin, and sectioned (5 μm thick). We used the Opal Polaris 7 Color Detection Kit (Akoya Biosciences, NEL861001KT) for multiplex immunofluorescent staining according to the manufacturer’s protocol. In brief, formalin-fixed paraffin-embedded (FFPE) slides were baked at 65 °C for 1 h, deparaffinized in xylene (3 × 10 min), rehydrated through degraded alcohols (100% ethanol, 3 × 5 min; 95% ethanol, 1 × 5 min; 75% ethanol, 1 × 5 min; and 50% ethanol, 1 × 5 min), briefly rinsed in distilled water, and fixed in 10% formalin for 20 min. After fixation, slides were briefly rinsed in water and placed in AR6 buffer (AR6001KT, provided in the Opal Polaris 7 Color Detection Kit). Heat-induced epitope retrieval was done with a 2100-Retriever, after which the slides were cooled down at room temperature for 30–60 min, rinsed in distilled water, followed by Tris-buffered saline with 0.05% Tween-20 (TBST). A hydrophobic barrier pen (Vector Laboratories, H-4000-2) was used to create a hydrophobic barrier around the tissue. Slides were then placed in a humidified chamber with blocking buffer (ARD10011EA, provided in the Opal Polaris 7 Color Detection Kit) at room temperature for 10 min. Next, slides were incubated with the primary antibody at room temperature for 1–2 h or at 4 °C overnight. Slides were washed in TBST (3 × 2 min) with agitation, followed by incubation in polymer HRP MS + Rb (ARH1001EA, provided in the Opal Polaris 7 Color Detection Kit) at room temperature for 10 min. Slides were washed again in TBST (3 × 2 min) with agitation, and the Opal Fluorophore working solution was applied to each slide and incubated at room temperature for 10 min. Slides were washed again in TBST (3 × 2 min) with agitation and then placed in AR6 buffer. Heat-induced epitope retrieval was performed again, and the protocol was repeated until all targets were detected. Slides were mounted with mounting medium with DAPI (Vector Laboratories, H-1200) and sealed with coverslips. The primary antibodies are as follows: anti-CD3 (1:200, Cell Signaling Technology, 99940S, RRID: AB_2755035) used with Opal480 (1:100), anti-CD8 (1:200, Cell Signaling Technology, 98941S, RRID: AB_2756376) used with Opal520 (1:100), and anti-granzyme B (1:200, Cell Signaling Technology, 44153 S, RRID: AB_2857976) used with Opal690 (1:100). The finished slides were scanned by the Vectra Polaris Automated Quantitative Pathology Imaging System and analyzed by Phenochart. The number of positive cells and the fluorescence intensity were quantitated by Image J software.

### CyTOF

CyTOF experiments were performed as described previously^53^. Briefly, tumor samples were dissociated on the gentleMACS Dissociator (Miltenyi Biotec) with the Mouse Tumor Dissociation Kit (Miltenyi Biotec, 130-096-730) and were depleted of red blood cells using RBC Lysis Buffer (BioLegend, 420301). 1 × 10^6^ cells per sample were used for staining. For dead cell staining, cells were incubated with cisplatin (2.5 μM, Fisher Scientific, NC0637801) for 1 min. Cells were Fc-blocked with an anti-CD16/CD32 antibody (BioLegend, 101320) for 10–20 min and then incubated with the CyTOF surface antibody mix for 30–60 min. Cells were incubated with 1.6% paraformaldehyde (PFA) in PBS for 30 min and incubated in cold 100% methanol at −20 °C overnight. For intracellular staining, cells were incubated with the CyTOF intracellular antibody mix for 30–60 min. For singlet discrimination, cells were washed and incubated with Cell-ID Intercalator-Ir (Fluidigm, 201192A) at 4 °C overnight. The samples were submitted to the Flow Cytometry and Cellular Imaging Core Facility at MD Anderson Cancer Center and run on CyTOF Instrumentation (DVS Science). CyTOF data were analyzed by Cytobank. Antibodies used for CyTOF are listed in Supplementary Table 1. Gating methods used for CyTOF analysis are listed in Supplementary Table 2.

### Cell culture

The male PDAC cell lines HY24409 and HY24160 and the female PDAC cell line HY19636 were established from KPC mice (p48-Cre;*Kras*^LSL-G12D/+^;*Trp53*^loxP/+^)^34^ that were backcrossed to a C57BL/6 background (purity: ~98%). The human PDAC cell lines SU86.86 (female), SW1990 (male), BXPC-3 (female), Miapaca-2 (male), Capan-1 (male), ASPC-1 (female), Hs766T (male), Colo357/FG (female), L3.6PL (female), Panc28 (female), Capan-2 (male), CF-Pac-1 (male), Panc3 (sex unknown), Panc1 (male), Panc48 (sex unknown), and HPAC (female) were from Mien-Chie Hung’s lab stock. The HY24409, HY24160, HY19636, SU86.86, SW1990, and Hs766T cell lines were cultured in RPMI 1640 (Corning, 10-041-CV) supplemented with 10% fetal bovine serum (FBS) (Gibco, 10438-034) and 1% penicillin/streptomycin (Sigma, P4333). The BXPC-3, Miapaca-2, Capan-1, ASPC-1, Colo357/FG, L3.6PL, Panc28, Capan-2, CF-Pac-1, Panc3, Panc1, Panc48, and HPAC cell lines were cultured in DMEM/F-12 (Corning, 10-092-CV) supplemented with 10% FBS and 1% penicillin/streptomycin. Cells were grown in a humidified incubator with 5% CO_2_ at 37 °C, and low-passage stocks were maintained in a centralized lab cell bank. Short tandem repeat profiling and mycoplasma tests were done by the Cytogenetics and Cell Authentication Core at MD Anderson Cancer Center.

For GR activation experiments, cells were cultured in phenol red-free RPMI 1640 (Gibco, 11835-030) supplemented with 5% charcoal-stripped FBS (Gibco, A33821-01) for 3 days, and then were cultured in the presence of dexamethasone (100 nM, Sigma, D2915) with or without mifepristone (100 nM, R&D, 1479/100) for 3–8 h.

### Lentiviral shRNA infection

For human GR knockdown, we used two shRNAs, TRCN0000245004 (shGR-1, 5′-GTGTCACTGTTGGAGGTTATT-3′) and TRCN0000245006 (shGR-2, 5′-TGGATAAGACCATGAGTATTG-3′) in the pLKO-puro vector from Sigma. Mouse GR knockdown was done with two shRNAs, TRCN0000026186 (5′-CCCAGAGATGTTAGCTGAAAT-3′) and TRCN0000238464 (5′-TGGATAAGTCCATGAGTATTG-3′), and mouse B2M knockdown was done with the shRNA TRCN0000295705 (5′-CCAGTTTCTAATATGCTATAC-3′), all in the pLKO-puro vector from Sigma. A non-targeting shRNA in the pLKO-puro backbone was used as the control. Lentivirus was produced by transfecting 4 μg lentiviral shRNA plasmid, 3 μg viral packaging plasmid pPAX2, and 1 μg envelope plasmid pMD2.G into HEK293T cells. Cells were infected in the presence of polybrene (5 μg ml^−1^, Sigma, TR-1003-G), and at 48–72 h after infection, cells were selected with 2 μg ml^−1^ puromycin (ThermoScientific, A1113803).

### Flow cytometry

For cultured cell lines, cells were incubated with the Accutase Cell Detachment Solution (BioLegend, 423201) and were washed twice with PBS. For immunophenotyping of tumors, tumor samples were dissociated, depleted of red blood cells, and Fc-blocked as described above. One million cells were stained with the Zombie Aqua Fixable Viability Kit (BioLegend, 423114). Cells were incubated with the indicated antibody diluted in staining buffer (2% FBS in PBS) at 4 °C in the dark for 30–60 min. Then, cells were washed twice and analyzed or further fixed in 1.6% PFA in PBS for 20 min. Intracellular staining was done with the Intracellular Staining Permeabilization Wash Buffer (BioLegend, 421002). Detection of cytokine production ex vivo was performed as described previously^44,54,55^. Tumor-infiltrating lymphocytes were enriched by Percoll (Fisher Scientific, 45-001-754) gradient centrifugation. Cells were resuspended in RPMI 1640 containing 10% FBS, stimulated by 50 ng ml^−1^ PMA (Sigma-Aldrich, P8139-1MG) and 3 μM ionomycin (R&D Systems, 1704/1) in the presence of 2.5 mg ml^−1^ Brefeldin A (BioLegend, 420601) at 37 °C for 4 h. Cells were processed for surface marker staining as described above. For intracellular cytokine staining, cells were fixed in Fixation Buffer (BioLegend, 420801) for 20 min and were washed two times with Permeabilization buffer (BioLegend, 421002). Cells were then stained with intracellular antibodies for 30 min. After staining, cells were analyzed on an Invitrogen Attune NxT Acoustic Focusing Cytometer and analyzed by FlowJo software. We gated tumor cells by ZombieDye^–^CD45^–^luciferase^+^, CTLs by ZombieDye^–^CD45^+^CD3^+^CD8^+^, and granzyme B-positive CTLs by ZombieDye^–^CD45^+^CD3^+^CD8^+^GB^+^. Gating strategies are shown in Supplementary Fig. 8. Antibodies used for flow cytometry are listed in Supplementary Table 3.

### Luciferase reporter assay

Human PD-L1 promoter-reporter constructs^33^ were purchased from Addgene (Addgene numbers: 107002, 107003, 107004, 107006, and 107007). The GR activity reporter plasmid was purchased from Qiagen (ID: C82DB0D7-3D10-4B1C-A358-411558D2DE01). The promoter regions of human *HLA-B*, *HLA-C*, and *B2M* were PCR-amplified from genomic DNA of the SU86.86 cell line (PCR primers are listed in Supplementary Table 4). The linearized pGL3-basic plasmid was amplified by PCR (forward primer: 5′-AGCTTGGCATTCCGGTACTG-3′, reverse primer: 5′-CTATCGATAGAGAAATGTTCTGGCACC-3′), and PCR products of promoter regions were ligated to linearized pGL3-basic using the In-Fusion HD Cloning Kit (Takara Bio, 638909).

The firefly luciferase reporter containing the human gene promoter was co-transfected with a Renilla luciferase vector (for normalization) into SU86.86 cells using jetPRIME transfection reagent (VWR, 89129-922). One day after transfection, cells were treated with IFNγ (10 ng ml^−1^, 8 h) or dexamethasone (100 nM, 8 h). Firefly and Renilla luciferase activities were measured using a Dual-Luciferase Reporter Assay (Promega, E1910) on a microplate reader according to the manufacturer’s protocol. Firefly luciferase activity was normalized to Renilla luciferase activity.

### Quantitative reverse-transcription PCR

Total RNA was extracted using TRIzol Reagent (Life technologies, 15596018) and the PuroLink RNA Mini Kit (Invitrogen, 12183018 A), and then reversed-transcribed with the iScript Reverse Transcription Supermix (Bio-Rad, 1708841). Quantitative PCR was performed with the iTaq Universal SYBR Green Supermix (Bio-Rad, 1725124) on a CFX96 real-time PCR machine (Bio-Rad). The mRNA level was calculated using the ΔCt method and normalized by *GAPDH*. Sequences for qPCR primers are listed in Supplementary Table 5.

### Immunoblotting

Cultured cells or homogenized mouse tissues were lysed in RIPA lysis buffer (Sigma-Aldrich, 20–188) containing protease inhibitors (GenDEPOT, P3100-001) and phosphatase inhibitors (GenDEPOT, P3200-001). Proteins were resolved on 4–12% (GenScript, M00654) or 4–20% (GenScript, M00657) precast gradient gels and transferred to a nitrocellulose membrane using the Trans-Blot Turbo Transfer System (Bio-Rad, 1704150). Membranes were blocked with 10% non-fat milk in TBST and incubated with the primary antibody at 4 °C overnight, followed by incubation with the secondary antibody conjugated with horseradish peroxidase (HRP). The bands were visualized with enhanced chemiluminescence substrate (ThermoFisher Scientific, 34578). Primary antibodies used are as follows: antibodies against GR (1:1000, Proteintech, 24050-1-AP, RRID: AB_2813890), phospho-GR (Ser211) (1:1000, Cell Signaling Technology, 4161S, RRID: AB_2155797), GAPDH (1:2000, Proteintech, 60004-1-IG, RRID: AB_2107436), PD-L1 (1:1000, Cell Signaling Technology, 13684S, RRID: AB_2687655), MHC-I (1:500, Santa Cruz Biotechnology, sc-55582, RRID: AB_831547), MHC-I (1:500, Santa Cruz Biotechnology, sc-32235, RRID: AB_627934), B2M (1:1000, Cell Signaling Technology, 12851S, RRID: AB_2716551), phospho-STAT1 (1:1000, Cell Signaling Technology, 9167S, RRID: AB_561284), STAT1 (1:1000, Cell Signaling Technology, 14994S, RRID: AB_2737027), and PR (1:1000, Proteintech, 25871-1-AP, RRID:AB_2880277). Immunoblotting images were obtained using the ChemiDoc Touch Imaging System (Bio-Rad) and Image Lab Touch software (Bio-Rad, version 2.3.0.07).

### Human samples and plasma cortisol measurement

The human tissue microarray and plasma samples were from the Cancer Hospital of the University of Chinese Academy of Sciences, Zhejiang Cancer Hospital (Hangzhou, China). All tissue and blood samples were collected with informed consent. The collection and use of human samples were approved by the Ethics Committee of Cancer Hospital of the University of Chinese Academy of Sciences, following the Declaration of Helsinki ethical guidelines. Human plasma cortisol levels were quantitated with an ELISA kit from ENZO Life Sciences (ADI-900-071) using blood samples collected at the same time (9 am local time).

### Immunohistochemical staining of tissue microarrays

IHC staining of tissue microarrays was done on the FFPE slides. After the xylene-alcohol-water workflow and the antigen retrieval step as described above, slides were incubated with blocking solution (Vector Laboratories, SP-6000-100) at room temperature for 10 min, followed by a quick wash with PBS. Slides were then incubated with 20% horse serum (Vector Laboratories, PK-7200) at room temperature for 20 min, followed by incubation with the primary antibody at 4 °C overnight. Slides were quickly rinsed with PBS and incubated with biotinylated universal secondary antibody (Vector Laboratories, PK-7200) or goat IgG HRP-conjugated antibody (R&D systems, HAF017) at room temperature for 30 min, followed by a quick rinse with PBS and incubation with ABC reagent (Vector Laboratories, SK-4100) for 30 min. DAB solution (Vector Laboratories, SK-4100) was applied at room temperature for 45 seconds, followed by counterstaining with hematoxylin QS (Vector 24 Laboratories, H-3404) at room temperature for 1 min, mounting (using medium: Vector Laboratories, H-5000-60), and sealing. Primary antibodies used for IHC are antibodies against GR (1:200, Sigma-Aldrich, SAB4501309; RRID: AB_10744954), PD-L1 (1:200, GeneTex, GTX01796), MHC-I (1:200, Santa Cruz Biotechnology, sc55582; RRID: AB_831547), and CD8 (1:100, MXB Biotechnologies, RMA-0514). In PDAC cells, PD-L1 and MHC-I were predominantly localized on the cell membrane, whereas GR was present in both the nucleus and the cytoplasm. Slides were scanned using a fully automated digital pathology slide system (KFBIO, KF-PRO-005). Histopathological review and IHC scoring were done by two pathologists (Wenjuan Yin and Weiya Xia). Positive and negative scores were assigned to GR and PD-L1. High and low scores were assigned to MHC-I, and expression was deemed high if > 20% of tumor cells were MHC-I positive. High and low scores were assigned to CD8, and expression was deemed high if > 10 cells per high-power field (×400) were positive.

### Chromatin immunoprecipitation

SU86.86 cells were grown to 40% confluence in 15-cm dishes in RPMI with 10%FBS, followed by 72-h incubation in phenol red-free RPMI supplemented with 5% charcoal-stripped FBS. Cells were treated with vehicle, 100 nM dexamethasone, or 100 nM dexamethasone + mifepristone for 30 min. We used the Simplechip® Plus Enzymatic Chromatin IP Kit (Magnetic Beads) (Cell Signaling Technology, 9005 S) for the following steps. Cells were cross-linked with 1% formaldehyde, quenched with glycine, and harvested. After cell lysis with ChIP lysis buffer, cells were digested with Micrococcal Nuclease and sonicated to achieve the majority of DNA fragments between 200 bp and 500 bp. GR was immunoprecipitated using 4 μg ChIP-grade rabbit anti-GR antibody (Proteintech, 24050-1-AP, RRID: AB_2813890), and 4 μg of rabbit IgG (Cell Signaling Technology, 2729) was used as a control. Chromatin was eluted from GR ChIP following the manufacturer’s protocol. GR-binding sites were predicted by PROMO (http://alggen.lsi.upc.es/cgi-bin/promo_v3/promo/promoinit.cgi?dirDB=TF_8.3). Primers for ChIP-qPCR are listed in Supplementary Table 6.

### Chemokine array

Chemokine array was performed with conditioned medium from HY24409 cells treated with vehicle or mifepristone (20 μM, 48 h) using the Proteome Profiler Mouse Chemokine Array Kit (R&D system, ARY020) following the manufacturer’s instructions. The mouse chemokine array coordinates are shown in Supplementary Table 7.

### Cell cycle analysis

Cells were seeded in six-well plates (2 × 10^5^ cells per well) and treated with 2 mM thymidine (Sigma-Aldrich, T9250-1G) for 18 h, fresh medium for 9 h, and 2 mM thymidine again for 17 h to arrest cells in G1/S phases for synchronization. Released cells were collected at the indicated time points by centrifugation, washed in cold PBS, resuspended in 1 ml of 70% ethanol, and stored at −20 °C overnight. Cells were then collected by centrifugation and washed two times with cold PBS. To ensure that only DNA was stained, we treated cells with 50 μl of 100 μg ml^−1^ RNase (New England Biolabs, T3018L), and added 425 μl of cell staining buffer (2% FBS in PBS) and 25 μl of propidium iodide solution (BioLegend, 421301). After staining, samples were analyzed by flow cytometry. Cells were gated for PI staining and the cells in G1, S, and G2/M phases were quantitated using FlowJo software.

### Computational data analysis

The correlation of expression levels of two genes was analyzed using the R corrplot package and the cor function. ICGC gene expression and clinical data were obtained from the International Cancer Genome Consortium data portal (https://dcc.icgc.org/repositories). Survival analysis was performed using the R survival package. Patient stratification was done using the R kmeans function on gene expression values.

### Statistics and reproducibility

Except for the animal studies (one time), chemokine array analysis (one time), and tissue microarray analysis (one time), each experiment was repeated at least three times with similar results. For qPCR assays of cell lines, we used *n* = 3 technical replicates per sample, and a representative set from three independent experiments is shown; source data for all three independent experiments are provided as a Source Data file. For all other experiments (including qPCR assays of mouse tissues and ChIP-qPCR assays), we used biological replicates. The statistical analysis for each plot was described in figure legends. Unless otherwise noted, data are presented as mean ± s.e.m, and Student’s *t*-test (two-tailed) was used to compare two groups of independent samples. The data analyzed by the *t*-test were normally distributed; we used an F-test to compare variances, and the variances were not significantly different. Therefore, when using a *t*-test, we assumed equal variance, and no data points were excluded from the analysis. *P* < 0.05 was considered statistically significant. Statistical analysis was performed using Graphpad Prism (GraphPad Software, version 8).

### Reporting summary

Further information on research design is available in the Nature Research Reporting Summary linked to this article.

## Supplementary information

Supplementary Information

Reporting Summary

## Data Availability

GR (encoded by *NR3C1*) mRNA levels in paired normal pancreatic tissue and PDAC were obtained from the dataset GSE15471 in the Gene Expression Omnibus. TCGA gene expression data were obtained from The Cancer Genome Atlas data portal (https://tcga-data.nci.nih.gov/tcga/dataAccessMatrix.htm). The source data that support the findings of this study are available with no restrictions. The uncropped blots are shown in Supplementary Fig. 9. Source data are provided with this paper.

## Source data

Source Data
