# Supplementary material for: Glucocorticoid receptor regulates PD-L1 and MHC-I in pancreatic cancer cells to promote immune evasion and immunotherapy resistance
Source: Nat Commun. 2021 Dec 6;12:7041. doi: 10.1038/s41467-021-27349-7 (PMC8649069; doi:10.1038/s41467-021-27349-7)
Supplement: Supplementary file 1 — Supplementary Information [file 41467_2021_27349_MOESM1_ESM.pdf]

Supplementary Information

Glucocorticoid receptor regulates PD-L1 and MHC-I in pancreatic cancer cells to promote immune evasion and immunotherapy resistance

Yalan Deng, Xianghou Xia, Yang Zhao, Zilong Zhao, Consuelo Martinez, Wenjuan Yin, Jun Yao, Qinglei Hang, Weiche Wu, Jie Zhang, Yang Yu, Weiya Xia, Fan Yao, Di Zhao, Yutong Sun, Haoqiang Ying, Mien-Chie Hung, Li Ma

Supplementary Fig. 1

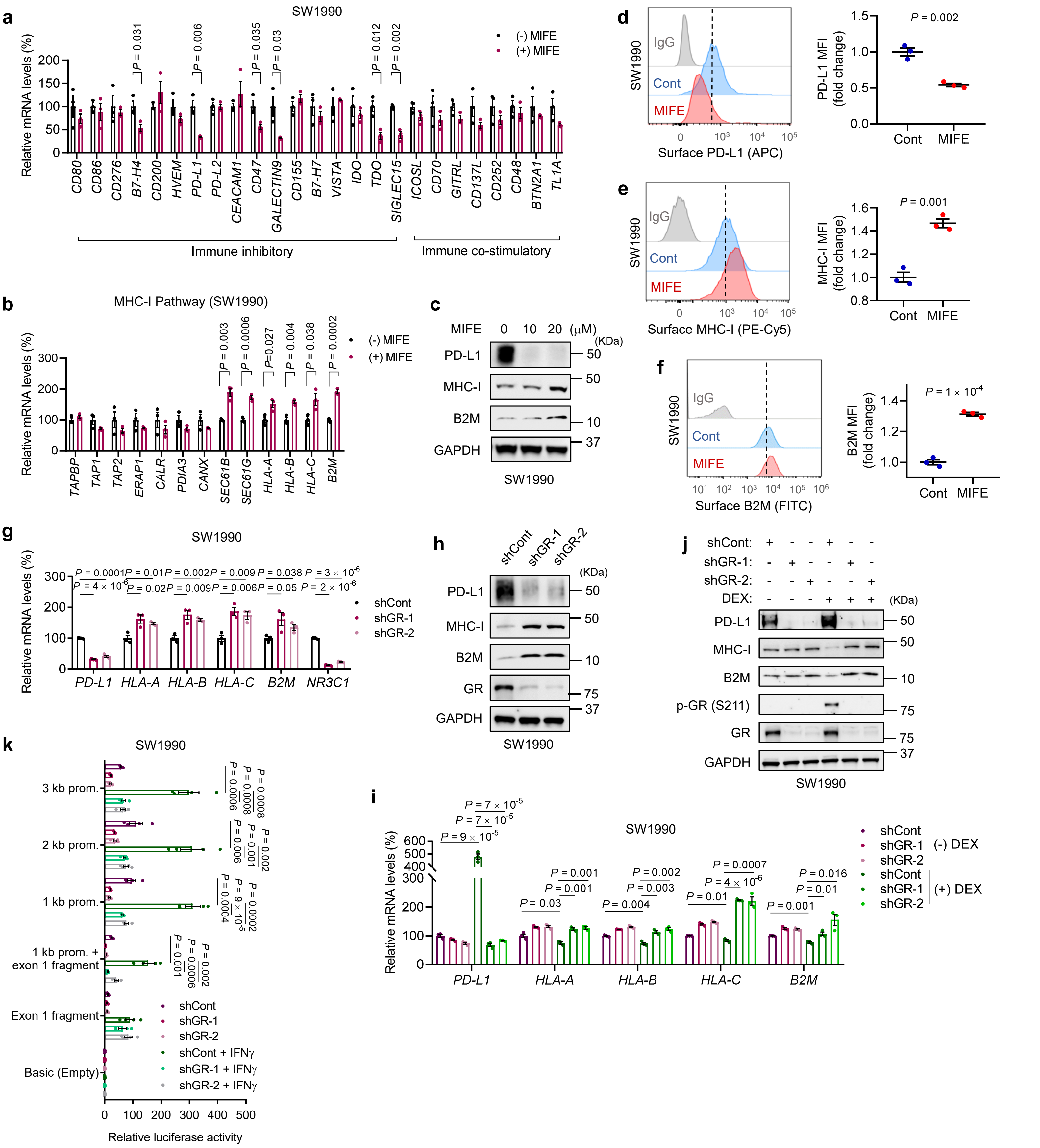

Supplementary Fig. 1. GR activates PD-L1 expression and represses MHC-I expression in the human PDAC cell line SW1990.

a and **b**, qPCR analysis of immune inhibitory and immune co-stimulatory genes (**a**) and genes involved in the MHC-I antigen presentation pathway (**b**) in SW1990 cells with or without mifepristone (MIFE, 20 μM, 72 h) treatment. $n = 3$ technical replicates per group.

c, Immunoblotting of PD-L1, MHC-I, and B2M in SW1990 cells with or without MIFE treatment with the indicated doses for 72 h.

d-**f**, Representative flow cytometry plots and quantification (by MFI: mean fluorescence intensity) of cell-surface PD-L1 (**d**), MHC-I (**e**), and B2M (**f**) in SW1990 cells with or without MIFE (20 μM, 72 h) treatment. $n = 3$ biological replicates per group.

g, qPCR analysis of *PD-L1*, *HLA-A*, *HLA-B*, *HLA-C*, *B2M*, and *NR3C1* in GR-knockdown SW1990 cells. $n = 3$ technical replicates per group.

h, Immunoblotting of PD-L1, MHC-I, B2M, and GR in GR-knockdown SW1990 cells.

i, qPCR analysis of *PD-L1*, *HLA-A*, *HLA-B*, *HLA-C*, and *B2M* in GR-knockdown SW1990 cells, with or without dexamethasone (DEX, 100 nM, 3 h) treatment. $n = 3$ technical replicates per group.

j, Immunoblotting of PD-L1, MHC-I, B2M, p-GR (Ser211), and GR in GR-knockdown SW1990 cells, with or without dexamethasone (DEX, 100 nM, 3 h) treatment.

k, Normalized luciferase activity of the reporters containing the indicated human *PD-L1* gene promoter regions in GR-knockdown SW1990 cells, with or without IFNγ (10 ng ml^{-1}, 8 h) treatment. $n = 4$ wells per group.

Statistical significance in **a**, **b**, **d-g**, **i**, and **k** was determined by a two-tailed unpaired *t*-test. Error bars are s.e.m. Source data are provided as a Source Data file.

Supplementary Fig. 2

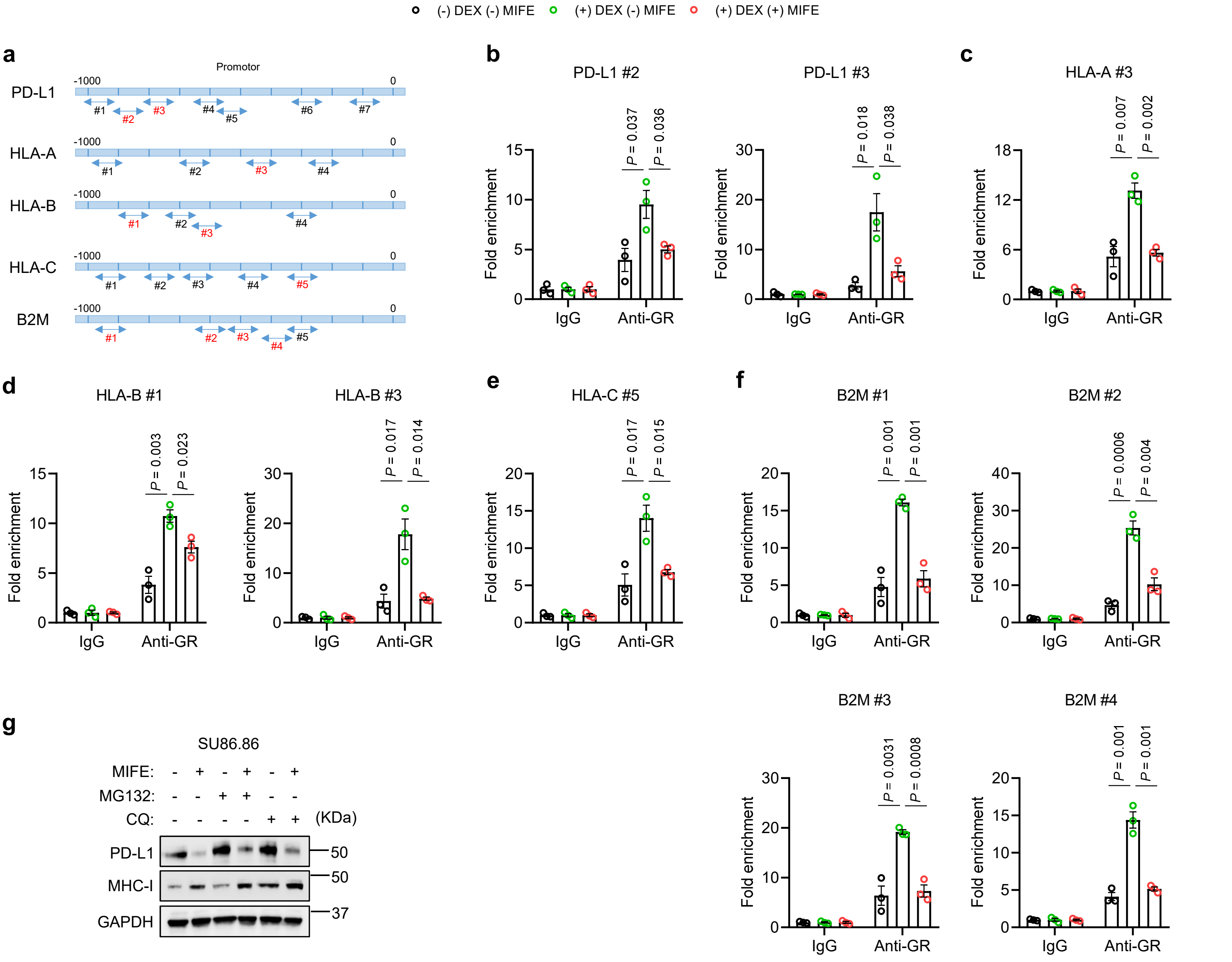

Supplementary Fig. 2. GR is a direct regulator of PD-L1 and MHC-I genes.

a, Schematic representation of human *PD-L1*, *HLA-A*, *HLA-B*, *HLA-C*, and *B2M* gene promoter regions and ChIP-qPCR amplicons. The red color indicates that the binding to GR was significantly induced by dexamethasone treatment, which was reversed by co-treatment with mifepristone.

b-**f**, ChIP-qPCR analysis showing the occupancy of *PD-L1* (**b**), *HLA-A* (**c**), *HLA-B* (**d**), *HLA-C* (**e**), and *B2M* (**f**) promoters by GR. Endogenous GR was immunoprecipitated from SU86.86 cells treated with vehicle or dexamethasone (100 nM) with or without mifepristone (100 nM) for 30 min. Statistical significance was determined by a two-tailed unpaired *t*-test. Error bars are s.e.m. $n = 3$ biological replicates per group.

g, Immunoblotting of PD-L1, MHC-I, and GAPDH in SU86.86 cells with or without the treatment of mifepristone (20 µM, 48 h), MG132 (10 µM, 6 h), and chloroquine (CQ, 20 µM, 4 h).

Source data are provided as a Source Data file.

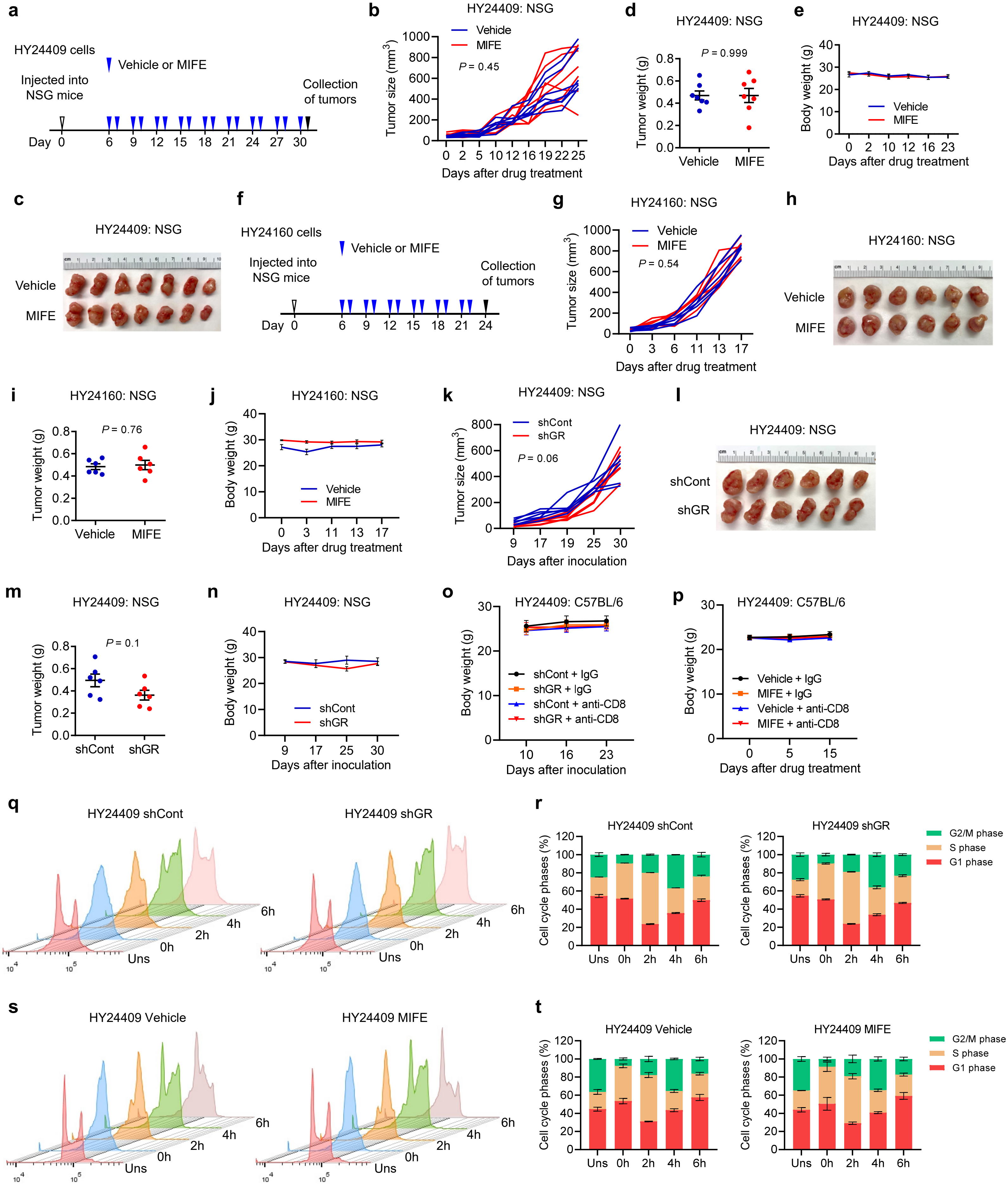
a
HY24409 cells
Vehicle or MIFE
Injected into NSG mice
Collection of tumors
Day 0 6 9 12 15 18 21 24 27 30
b
HY24409: NSG
Vehicle
MIFE
P = 0.45
Tumor size (mm3)
Days after drug treatment
d
HY24409: NSG
P = 0.999
Tumor weight (g)
Vehicle
MIFE
e
HY24409: NSG
Body weight (g)
Vehicle
MIFE
Days after drug treatment
c
HY24409: NSG
Vehicle
MIFE
f
HY24160 cells
Vehicle or MIFE
Injected into NSG mice
Collection of tumors
Day 0 6 9 12 15 18 21 24
g
HY24160: NSG
Vehicle
MIFE
P = 0.54
Tumor size (mm3)
Days after drug treatment
h
HY24160: NSG
Vehicle
MIFE
i
HY24160: NSG
P = 0.76
Tumor weight (g)
Vehicle
MIFE
j
HY24160: NSG
Body weight (g)
Vehicle
MIFE
Days after drug treatment
k
HY24409: NSG
shCont
shGR
P = 0.06
Tumor size (mm3)
Days after inoculation
l
HY24409: NSG
shCont
shGR
m
HY24409: NSG
P = 0.1
Tumor weight (g)
shCont
shGR
n
HY24409: NSG
Body weight (g)
shCont
shGR
Days after inoculation
o
HY24409: C57BL/6
Body weight (g)
shCont + IgG
shGR + IgG
shCont + anti-CD8
shGR + anti-CD8
Days after inoculation
p
HY24409: C57BL/6
Body weight (g)
Vehicle + IgG
MIFE + IgG
Vehicle + anti-CD8
MIFE + anti-CD8
Days after drug treatment
q
HY24409 shCont
HY24409 shGR
Uns 0h 2h 4h 6h
r
HY24409 shCont
HY24409 shGR
Cell cycle phases (%)
G2/M phase
S phase
G1 phase
Uns 0h 2h 4h 6h
s
HY24409 Vehicle
HY24409 MIFE
Uns 0h 2h 4h 6h
t
HY24409 Vehicle
HY24409 MIFE
Cell cycle phases (%)
G2/M phase
S phase
G1 phase
Uns 0h 2h 4h 6h

Supplementary Fig. 3. GR inhibition or depletion does not affect cell cycle progression or tumor growth in immunodeficient mice.

a-e, Tumor-bearing NSG mice (implanted with 4×10^4 HY24409 cells) received vehicle or mifepristone (MIFE) treatment. n = 7 mice per group.

a, Study design.

b, Tumor growth curves.

c, Endpoint tumor images.

d, Endpoint tumor weight.

e, Body weight.

f-j, Tumor-bearing NSG mice (implanted with 8×10^4 HY24160 cells) received vehicle or mifepristone (MIFE) treatment. n = 6 mice per group.

f, Study design.

g, Tumor growth curves.

h, Endpoint tumor images.

i, Endpoint tumor weight.

j, Body weight.

k-n, NSG mice were implanted with 1×10^5 HY24409 cells transduced with control shRNA or GR shRNA. n = 6 mice per group.

k, Tumor growth curves.

l, Endpoint tumor images.

m, Endpoint tumor weight.

n, Body weight.

o and **p**, Body weight of C57BL/6 mice bearing orthotopic HY24409 tumors expressing either control shRNA or GR shRNA (**o**), and C57BL/6 mice bearing vehicle- and MIFE-treated orthotopic HY24409 tumors (**p**), with or without $CD8^+$ T cell depletion. n = 5 mice per group.

q and **r**, Cell-cycle profile histograms (**q**) and quantitative analysis of the cell-cycle distribution (**r**) in HY24409 cells transduced with control shRNA or GR shRNA. Cells were collected from unsynchronized (Uns) conditions and at different time points after release from double thymidine block. n = 3 biological replicates per group.

s and **t**, Cell-cycle profile histograms (**s**) and quantitative analysis of the cell-cycle distribution (**t**) in HY24409 cells pretreated with vehicle or mifepristone (20 μM, 48 h), followed by double-thymidine block and release. n = 3 biological replicates per group.

Statistical significance was determined by two-way analysis of variance in **b**, **g**, and **k**, and was determined by a two-tailed unpaired t-test in **d**, **i**, and **m**. Error bars are s.e.m. Source data are provided as a Source Data file.

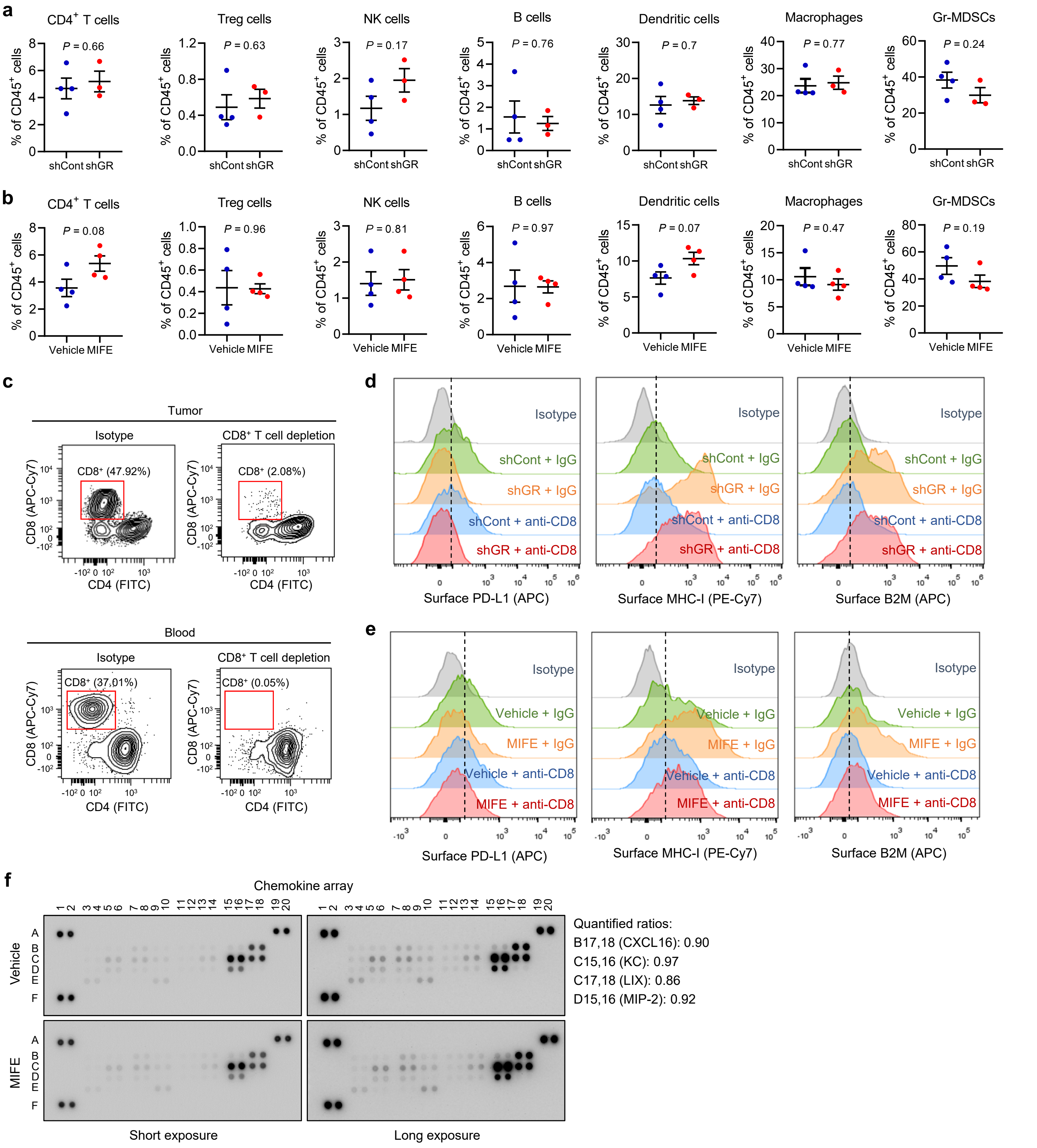
a
CD4+ T cells
Treg cells
NK cells
B cells
Dendritic cells
Macrophages
Gr-MDSCs
% of CD45+ cells
P = 0.66
P = 0.63
P = 0.17
P = 0.76
P = 0.7
P = 0.77
P = 0.24
shCont shGR
b
P = 0.08
P = 0.96
P = 0.81
P = 0.97
P = 0.07
P = 0.47
P = 0.19
Vehicle MIFE
c
Tumor
Isotype
CD8+ T cell depletion
CD8+ (47.92%)
CD8+ (2.08%)
CD8 (APC-Cy7)
CD4 (FITC)
Blood
CD8+ (37.01%)
CD8+ (0.05%)
d
Isotype
shCont + IgG
shGR + IgG
shCont + anti-CD8
shGR + anti-CD8
Surface PD-L1 (APC)
Surface MHC-I (PE-Cy7)
Surface B2M (APC)
e
Vehicle + IgG
MIFE + IgG
Vehicle + anti-CD8
MIFE + anti-CD8
f
Chemokine array
Vehicle
MIFE
Short exposure
Long exposure
Quantified ratios:
B17,18 (CXCL16): 0.90
C15,16 (KC): 0.97
C17,18 (LIX): 0.86
D15,16 (MIP-2): 0.92

Supplementary Fig. 4. Effects of GR depletion or inhibition on mice bearing the KPC line HY24409.

a, Quantification of tumor-infiltrating immune cell populations in orthotopic HY24409 tumors expressing either control shRNA or GR shRNA. Gr-MDSCs: granulocytic myeloid-derived suppressor cells. n = 3-4 mice per group.

b, Quantification of tumor-infiltrating immune cell populations in vehicle- and mifepristone (MIFE)-treated orthotopic HY24409 tumors. n = 4 mice per group. Statistical significance in **a** and **b** was determined by a two-tailed unpaired t-test. Error bars are s.e.m.

c, Representative flow cytometry plots (pre-gated on T cells by $CD45^{+}CD3^{+}$) showing the relative abundance of $CD8^{+}$ T cells in HY24409 tumor-bearing mice that received isotype control (IgG) or anti-CD8 antibody treatment. Flow cytometry was performed on tumor samples (upper panel) and blood samples (lower panel).

d, Representative flow cytometry plots of cell-surface PD-L1 (left), MHC-I (H-$2K^{b}$) (middle), and B2M (right) levels in cancer cells (gated by $ZombieDye^{-}CD45^{-}luciferase^{+}$) from orthotopic HY24409 tumors expressing either control shRNA or GR shRNA, with or without $CD8^{+}$ T cell depletion. Related to **Fig. 4j**.

e, Representative flow cytometry plots of cell-surface PD-L1 (left), MHC-I (H-$2K^{b}$) (middle), and B2M (right) levels in cancer cells (gated by $ZombieDye^{-}CD45^{-}luciferase^{+}$) from vehicle- and MIFE-treated orthotopic HY24409 tumors, with or without $CD8^{+}$ T cell depletion. Related to **Fig. 4k**.

f, Chemokine arrays of the conditioned medium of HY24409 cells with or without MIFE (20 μM, 48 h) treatment. See also **Supplementary Table 7**.

Source data are provided as a Source Data file.

Supplementary Fig. 5

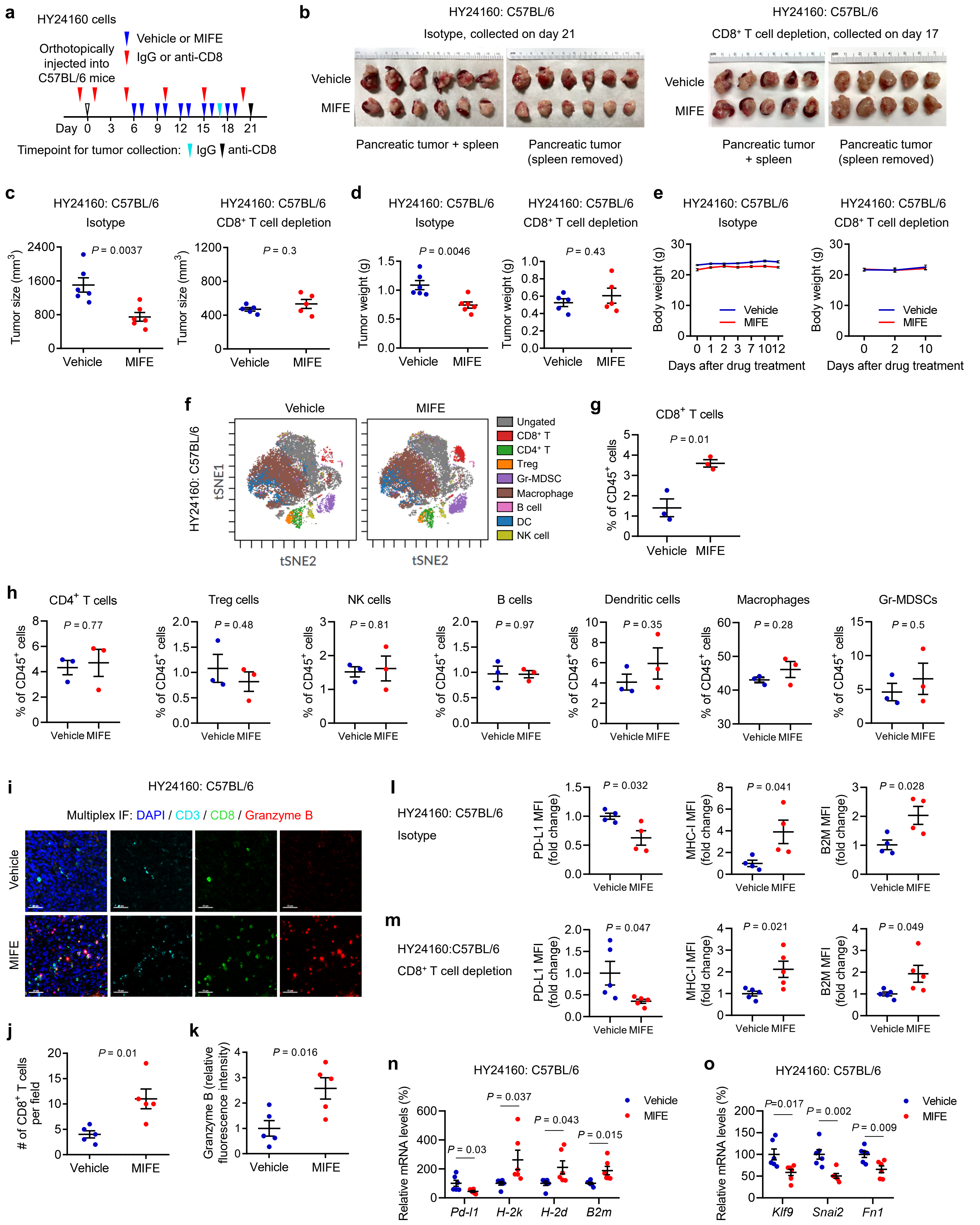

Supplementary Fig. 5. Effects of GR inhibition on anti-tumor immunity in the KPC line HY24160.

a-o, C57BL/6 mice bearing orthotopic pancreatic tumors (implanted with 4×10^4 HY24160 cells) received vehicle or mifepristone (MIFE) and isotype control (IgG) or anti-CD8 antibody treatment. *n* = 5-6 mice per group.

a, Study design.

b, Endpoint tumor images.

c, Endpoint tumor size.

d, Endpoint tumor weight.

e, Body weight.

f, Immune profiling of vehicle- and MIFE-treated orthotopic HY24160 tumors by CyTOF. Representative viSNE plots were colored by immune cell populations.

g, $CD8^+$ T cell quantification.

h, Quantification of tumor-infiltrating immune cell populations in vehicle- and MIFE-treated orthotopic HY24160 tumors. Gr-MDSCs: granulocytic myeloid-derived suppressor cells.

i-k, Multiplex immunofluorescent staining of CD3, CD8, and granzyme B in vehicle- and MIFE-treated orthotopic HY24160 tumors (**i**), and quantification of CD8 (**j**) and granzyme B (**k**) signals. Scale bars, 50 µm.

l and **m**, Flow cytometric analysis of cell-surface PD-L1 and MHC-I ($H\text{-}2K^b$) levels in tumor cells collected from mice treated with isotype control IgG (**l**) or anti-CD8 antibody (**m**). MFI: mean fluorescence intensity.

n, qPCR analysis of *Pd-l1*, *H-2k*, *H-2d*, and *B2m* in vehicle- and MIFE-treated orthotopic HY24160 tumors.

o, qPCR analysis of known GR-activated target genes (*Klf9*, *Snai2*, and *Fn1*) in vehicle- and MIFE-treated orthotopic HY24160 tumors.

Statistical significance in **c**, **d**, **g**, **h**, and **j-o** was determined by a two-tailed unpaired *t*-test. Error bars are s.e.m. Source data are provided as a Source Data file.

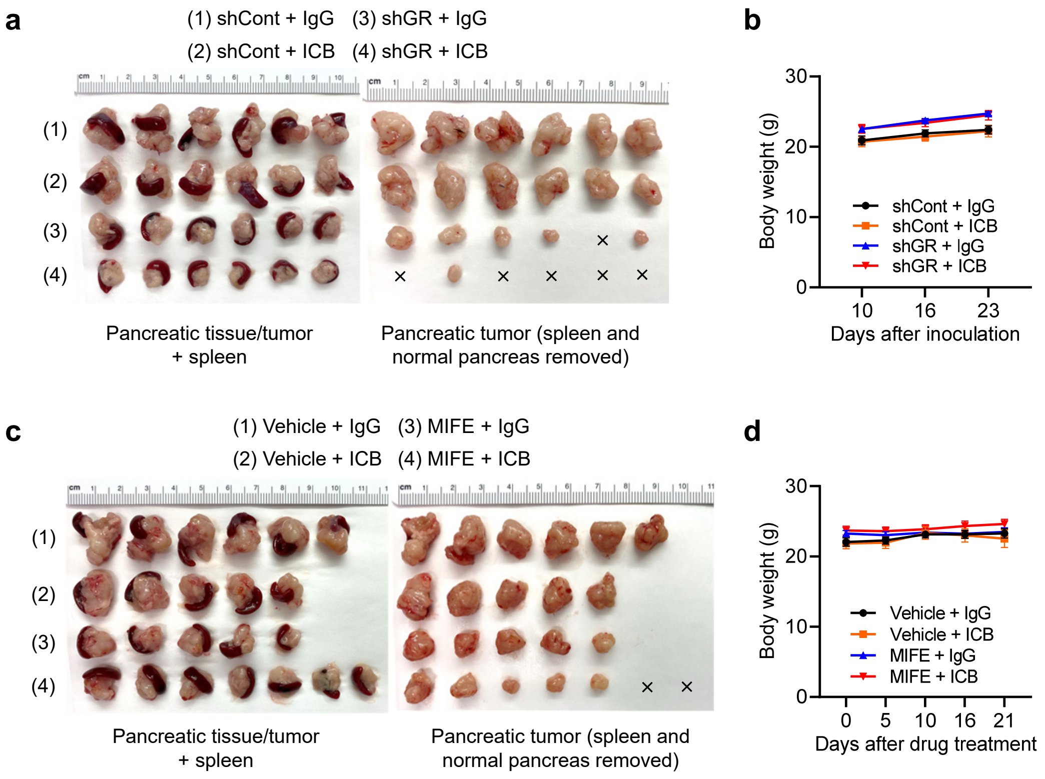

Supplementary Fig. 6. Effect of GR depletion or inhibition on the immunotherapeutic response of the KPC line HY24409.

a, Endpoint images of orthotopic HY24409 tumors expressing either control shRNA or GR shRNA, with or without dual ICB (anti-PD1 and anti-CTLA-4 monoclonal antibodies) treatment. n = 6 mice per group. Related to **Fig. 6d**.

b, Body weight of the mice described in **a**.

c, Endpoint images of orthotopic HY24409 tumors from C57BL/6 mice treated with mifepristone (MIFE) and dual ICB (anti-PD1 and anti-CTLA-4 monoclonal antibodies), alone or in combination. n = 5-7 mice per group. Related to **Fig. 6h**.

d, Body weight of the mice described in **c**.

Source data are provided as a Source Data file.

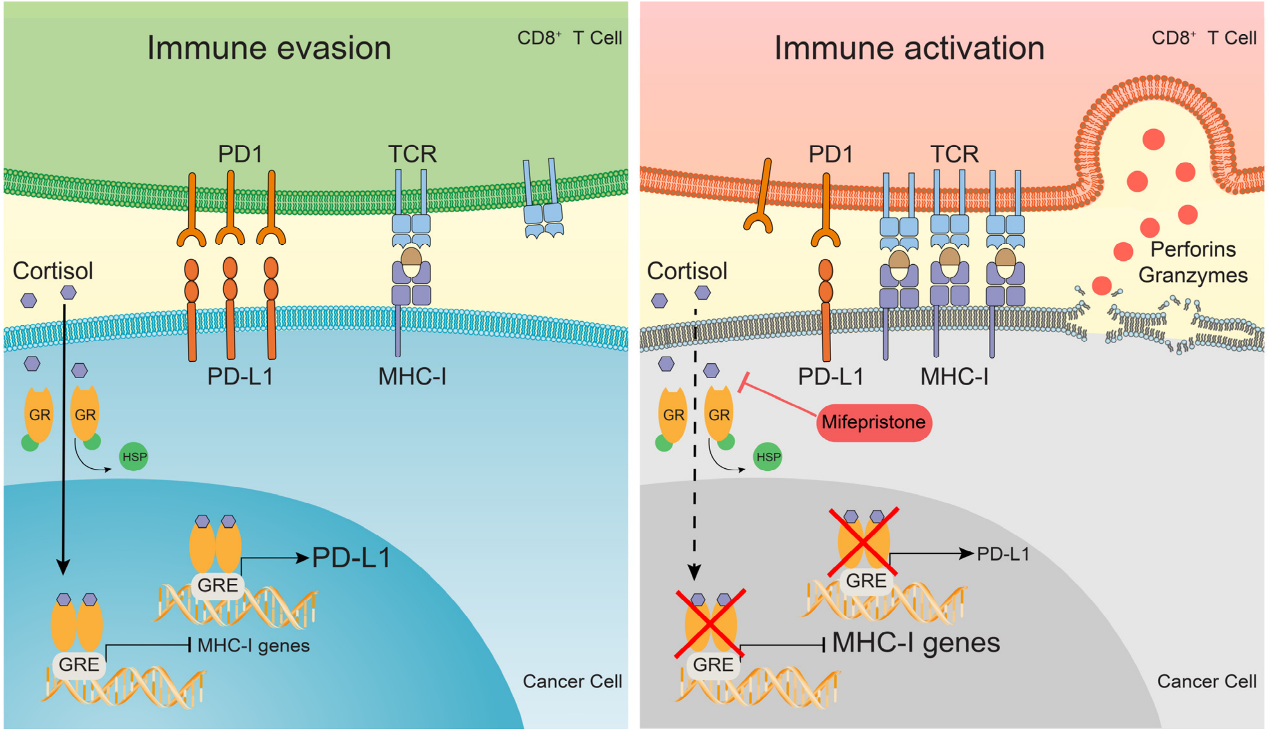

Supplementary Fig. 7. Model for the immunosuppressive effects of tumoral GR on the tumor microenvironment of pancreatic cancer. PD-1: programmed death-1. PD-L1: programmed death ligand-1. TCR: T cell receptor. MHC-I: major histocompatibility complex class I. HSP: heat shock protein. GRE: glucocorticoid response element.

Supplementary Fig. 8

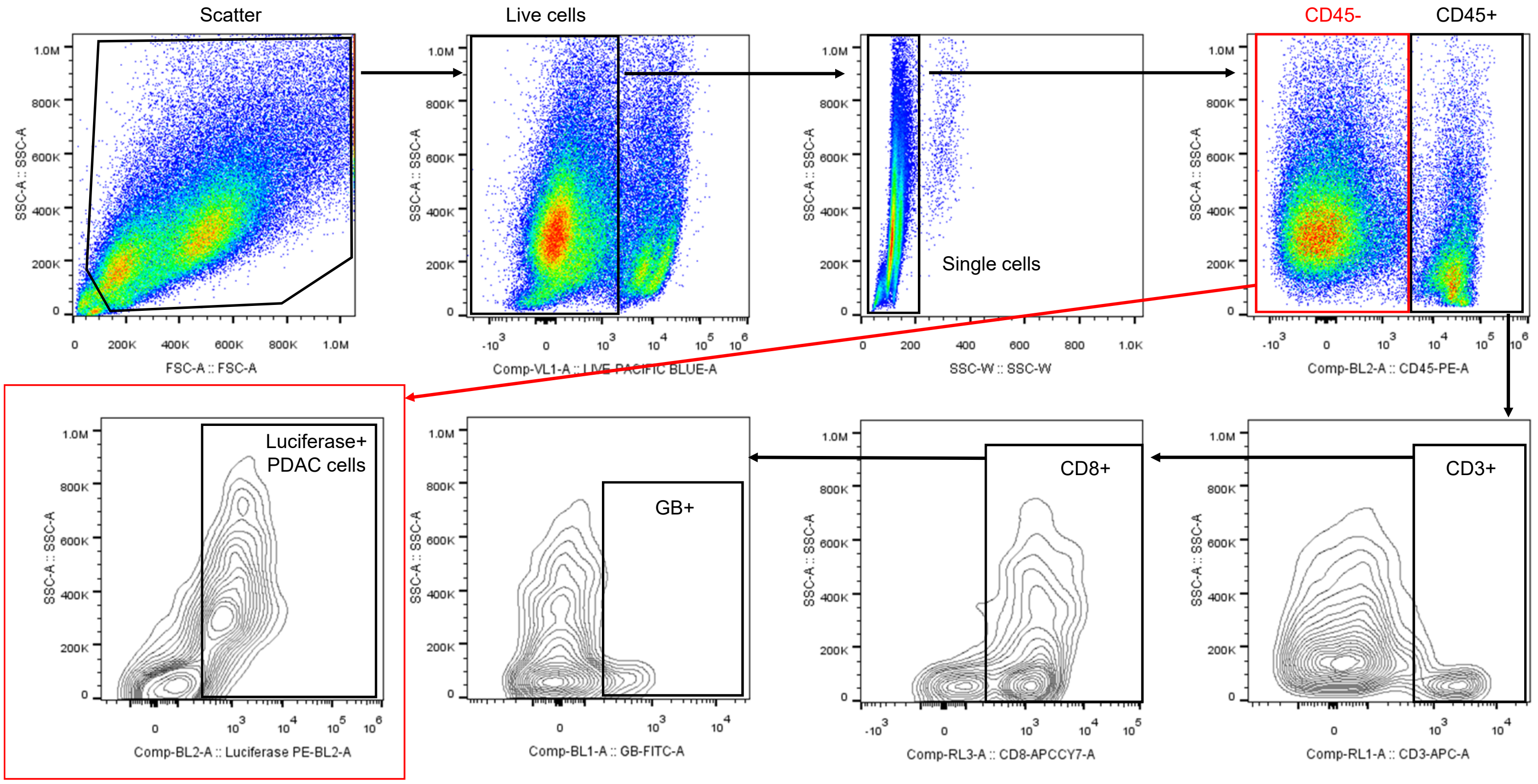

Supplementary Fig. 8. Gating strategies for flow cytometry analysis.

Figure 1c

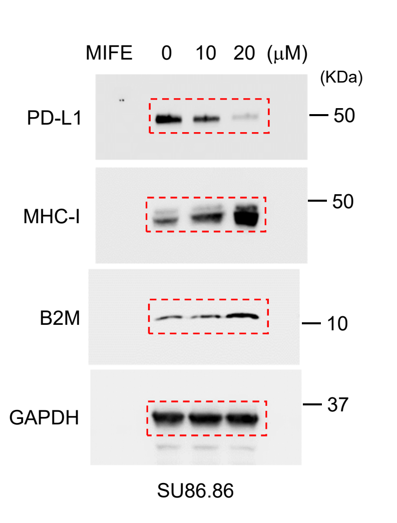

Figure 1h

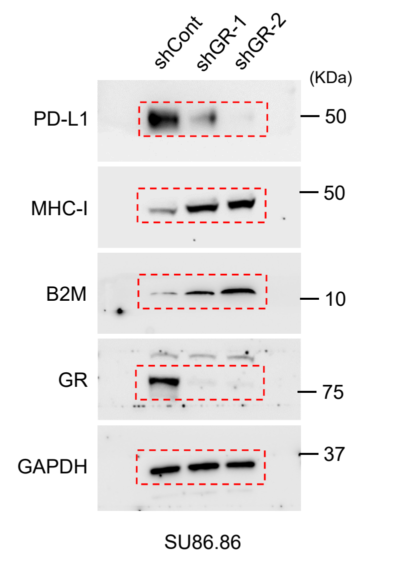

Figure 1m

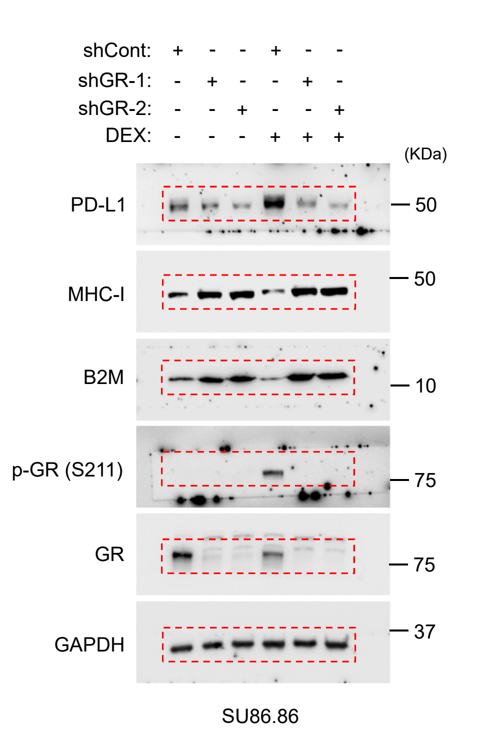

Figure 2a

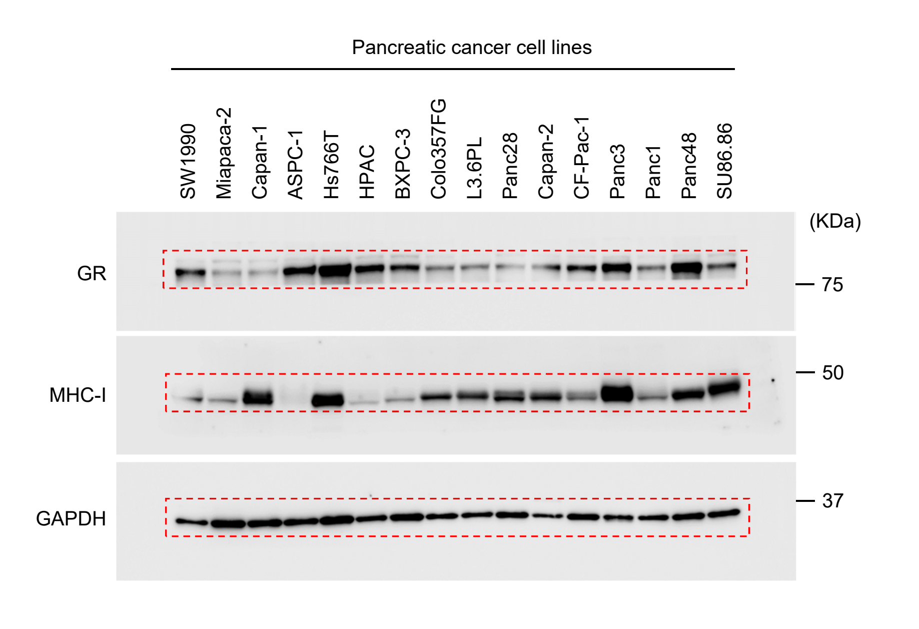

Figure 2f

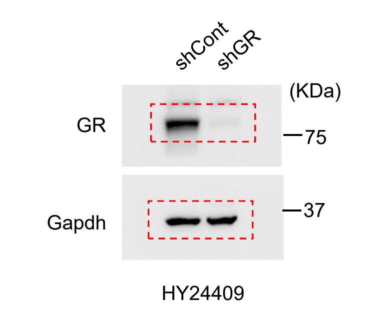

Figure 2d

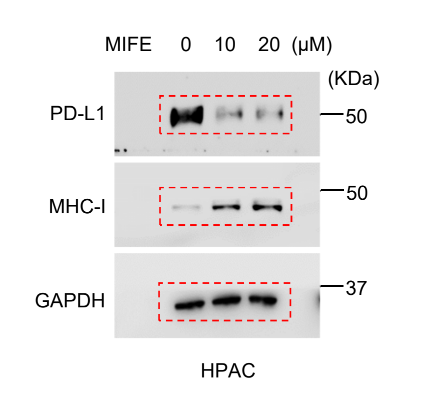

Figure 2e

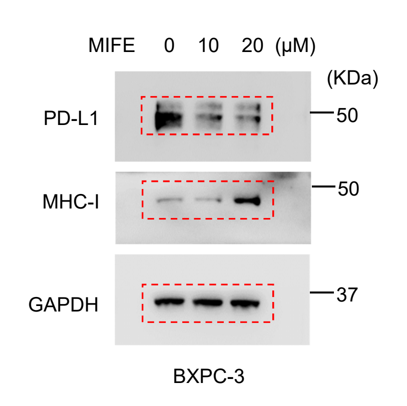

Figure 2l

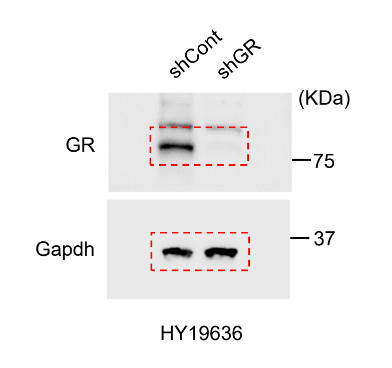

Supplementary Fig. 9. All uncropped blots in this manuscript.

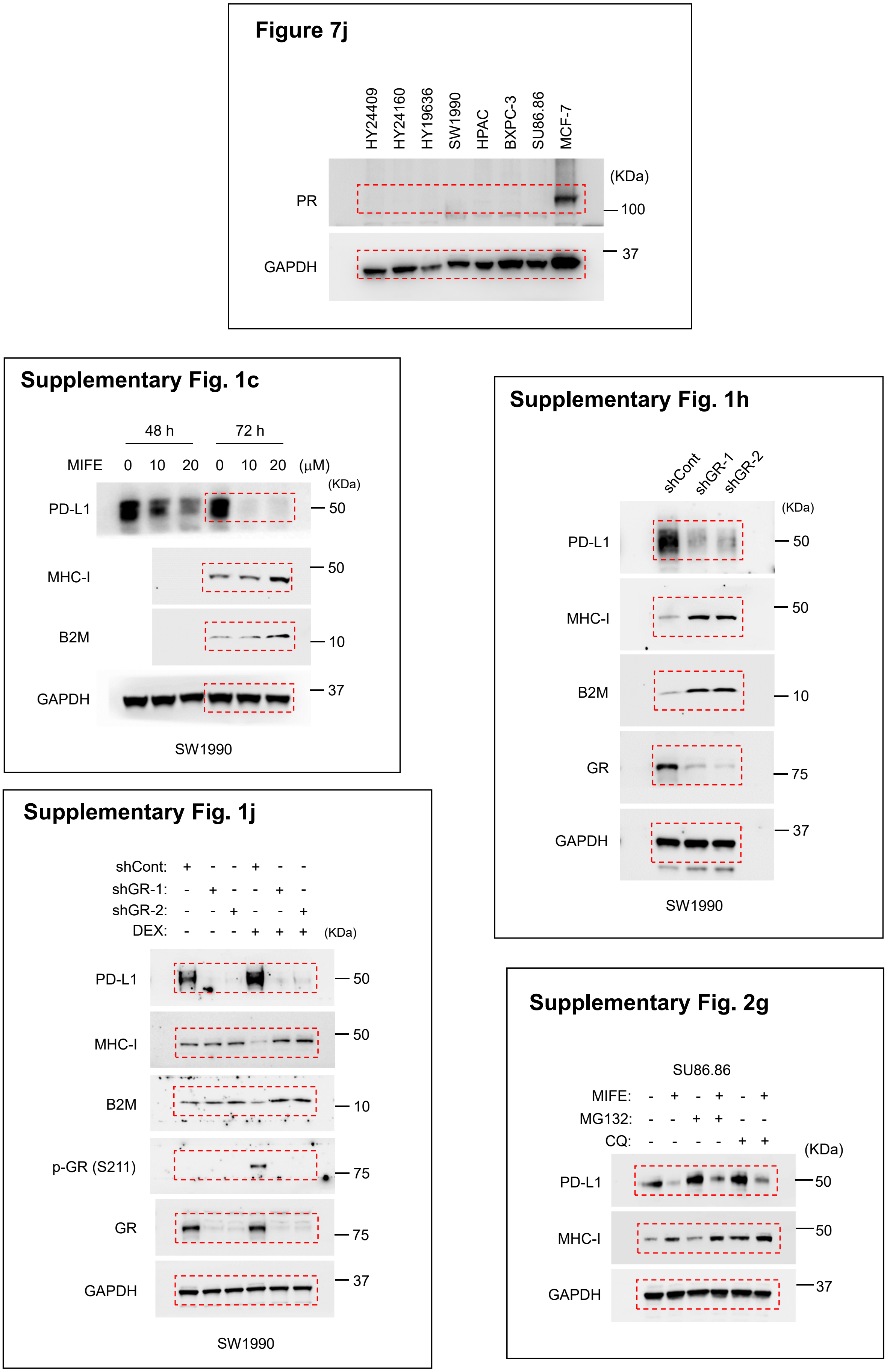

Supplementary Fig. 9. All uncropped blots in this manuscript.

Supplementary Tables

Supplementary Table 1. Antibodies used for CyTOF analysis

For HY24409 tumors:

Target	Label	Intracellular staining	Clone#	Dilution ratio	Source	Catalog#
CD11b	139La	FALSE	M1/70	1:500	BioLegend	101249
CD11c	142Nd	FALSE	N418	1:400	BioLegend	117302
CD19	149Sm	FALSE	4D5	1:400	BioLegend	115502
CD25	150Nd	FALSE	3C7	1:200	BioLegend	101902
CD3, CD3e	152Sm	FALSE	145-2C11	1:500	BioLegend	100302
CD4(Ms)	115In	FALSE	RM4-5	1:500	BioLegend	100506
CD45(Ms)	89Y	FALSE	30-F11	1:400	DVS-Fluidigm	3089005B
CD8a	146Nd	FALSE	53-6.7	1:500	BioLegend	100702
F4/80	171Yb	FALSE	D2S9R	1:400	Cell Signaling Technology	70076BF
Foxp3	158Gd	TRUE	FJK-16s	1:500	DVS-Fluidigm	3158003A
I-A/I-E, MHC-II	209Bi	FALSE	M5/114.15.2	1:1000	DVS-Fluidigm	3209006B
Ly-6G/Ly-6C, Gr-1	141Pr	FALSE	RB6-8C5	1:800	BioLegend	108402
NK1.1	170Er	FALSE	PK136	1:400	BioLegend	108702

For HY24160 tumors:

Target	Label	Intracellular staining	Clone	Dilution ratio	Source	Catalog#
CD45(Ms)	89Y	FALSE	30-F11	1:400	DVS-Fluidigm	3089005B
CD3, CD3e	152Sm	FALSE	145-2C11	1:500	BioLegend	100302
CD8a	146Nd	FALSE	53-6.7	1:500	BioLegend	100702
CD4(Ms)	115In	FALSE	RM4-5	1:500	BioLegend	100506
F4/80	173Yb	FALSE	BM8	1:400	BioLegend	123102
CD11b	139La	FALSE	M1/70	1:500	BioLegend	101249
Ly-6C	162Dy	FALSE	HK1.4	1:600	DVS-Fluidigm	3162014B
Ly-6G	141Pr	FALSE	1A8	1:400	DVS-Fluidigm	3141008B
CD25	150Nd	FALSE	3C7	1:200	BioLegend	101902
Foxp3	158Gd	TRUE	FJK-16s	1:500	DVS-Fluidigm	3158003A
CD19	149Sm	FALSE	4D5	1:400	BioLegend	115502
CD11c	142Nd	FALSE	N418	1:400	BioLegend	117302
NK1.1	170Er	FALSE	PK136	1:400	BioLegend	108702
I-A/I-E, MHC-II	209Bi	FALSE	M5/114.15.2	1:1000	DVS-Fluidigm	3209006B

Supplementary Table 2. Gating methods used for CyTOF analysis

$CD8^+$ T cells	$CD45^+CD11b^-CD3^+CD8^+CD4^-$
$CD4^+$ T cells	$CD45^+CD11b^-CD3^+CD8^-CD4^+$
Treg cells	$CD45^+CD11b^-CD3^+CD8^-CD4^+Foxp3^+$
Gr-MDSCs	$CD45^+CD11b^+CD3^-Gr1^+$
Macrophages	$CD45^+CD11b^+CD3^-Gr1^-F4/80^+$
B cells	$CD45^+CD11b^+CD3^-CD19^+$
Dendritic cells	$CD45^+CD11b^+CD3^-MHC\text{-}II^+CD11c^+$
NK cells	$CD45^+CD11b^+CD3^-NK1.1^+$

Supplementary Table 3. Antibodies for flow cytometry

Target	Color	Intracellular Staining	Clone#	Specificity	Dilution ratio	Source	Catalog#
PD-L1	APC	FALSE	10F.9G2	Mouse	1:80	BioLegend	124312
CD45	PE	FALSE	30-F11	Mouse	1:100	BioLegend	103106
CD45	FITC	FALSE	30-F11	Mouse	1:100	BioLegend	103108
CD3	APC	FALSE	145-2C11	Mouse	1:40	BioLegend	100312
CD8	APC-Cy7	FALSE	53-6.7	Mouse	1:40	BioLegend	100714
CD4	FITC	FALSE	GK1.5	Mouse	1:80	BioLegend	100406
B2M	APC	FALSE	A16041A	Mouse	1:40	BioLegend	154506
B2M	PE	FALSE	A16041A	Mouse	1:40	BioLegend	154504
H-2K^b	PE-Cy7	FALSE	AF6-88.5	Mouse	1:80	BioLegend	116520
H-2K^b	APC	FALSE	AF6-88.5	Mouse	1:80	BioLegend	116518
H-2K^b/D^b	-	FALSE	28-8-6	Mouse	1:200	BioLegend	114602
Luciferase	PE	TRUE	Luci 21 1-107	-	1:200	Novus Biologicals	NB600-307PE
HLA-A/B/C	PE	FALSE	W6/32	Human	1:40	BioLegend	311406
HLA-A/B/C	PE-Cy5	FALSE	G46-2.6	Human	1:10	BD	555554
B2M	FITC	FALSE	2M2	Human	1:40	BioLegend	316304
PD-L1	APC	FALSE	29E.2A3	Human	1:40	BioLegend	329708
IL-2	PE	TRUE	JES6-5H4	Mouse	1:40	BioLegend	503807
TNFα	PE	TRUE	MP6-XT22	Mouse	1:80	BioLegend	506305
IFNγ	PE-Cy7	TRUE	XMG1.2	Mouse	1:40	BioLegend	505825
PD-1	PE-Cy7	FALSE	29F.1A12	Mouse	1:80	BioLegend	135215
LAG-3	PE	FALSE	C9B7W	Mouse	1:40	BioLegend	125207
Tim-3	PE	FALSE	RMT3-23	Mouse	1:80	BioLegend	119703

Supplementary Table 4. Primers for luciferase reporter plasmid construction

Gene name	Forward primer (5' to 3')	Reverse primer (5' to 3')
B2M	tttctctatcgataggtcatgtttgtagacttcccaaatttgcc	ccggaatgccaagctaacttggagaagggaagtcacgg
HLA-B	tttctctatcgataggcgcagctaaaactggtgaat	ccggaatgccaagctgcgtctgaggagactctgagt
HLA-C	tttctctatcgatagaaacccataaaattcatttcctaggggt	ccggaatgccaagctgagagcagcaggaggaggg

Supplementary Table 5. Primers for qPCR

For human genes:

Gene name	Forward primer (5' to 3')	Reverse primer (5' to 3')
CALR	TCAAGGAGCAGTTTCTGGACGG	GCATCCTGGCTTGTCTGCAAAC
CANX	GCTGGTTAGATGATGAGCCTGAG	ACACCACATCCAGGAGCTGACT
ERAP1	GTCTGTCAGTGTGACCCATCCT	CTGAGCAGGATTTTCCACAGGTG
PDIA3	GTCAGCCACTTGAAGAAGCAGG	TAGGAACTCGGAGTGAGCCTCA
TAPBP	GAGCCTGTTCTCATCACCATGG	GTAGGCAAAGCTCAAGTCCAGC
TAP1	GCAGTCAACTCCTGGACCACTA	CAAGGTTCCCACTGCTTACAGC
TAP2	ATGCCCTTCACAATAGCAGCGG	CCAAAACTGCGAACGGTCTGCA
SEC61B	CCGCACAACCTCGGCAGGCA	CAGAAGCGATGAACAGAAGACTC
SEC61G	GCAGTTTGTTGAGCCAAGTCG	CCAGCCGAATGGAGTCCTT
HLA-A	AGATACACCTGCCATGTGCAGC	GATCACAGCTCCAAGGAGAACC
HLA-B	CTGCTGTGATGTGTAGGAGGAAG	GCTGTGAGAGACACATCAGAGC
HLA-C	GGAGACACAGAAGTACAAGCGC	ACATCCTCTGGAGGGTGTGAGA
B2M	CCACTGAAAAAGATGAGTATGCCT	CCAATCCAAATGCGGCATCTTCA
GAPDH	AAGGTGAAGGTCGGAGTCAAC	GAAGGGGTCATTGATGGCAAC
CD86	CCATCAGCTTGTCTGTTTCATTCC	GCTGTAATCCAAGGAATGTGGTC
ICOSL	GTTTCACTGCCTGGTGTTGAGC	ACGACGGGCACGCTGAAGTTTG
CD276	CTGGCTTTCGTGTGCTGGAGAA	GCTGTCAGAGTGTTTCAGAGGC
B7H4	CTCACAGATGCTGGCACCTACA	GCAAGGTCTCTGAGCTGGCATT
CD200	CCTGGAGGATGAAGGGTGTTAC	AGTGAAGGGATACTATGGGCTGT
CD70	TTCGCACAGGCTCAGCAGCAG	TTGTCCAGCTCTGGTCCATGCA
CD274	TGCCGACTACAAGCGAATTACTG	CTGCTTGTCCAGATGACTTCGG
PDL2	CTCGTTCCACATACCTCAAGTCC	CTGGAACCTTTAGGATGTGAGTG
CD155	CACTGTCACCAGCCTCTGGATA	TCATAGCCAGAGATGGATACCTC
GALECTIN9	ACACCCAGATCGACAACTCCTG	CAAACAGGTGCTGACCATCCAC
CD47	TATCCTCGCTGTGGTTGGACTG	TAGTCCAAGTAATTGTGCTAGAGC
CEACAM1	CACGCCAATAACTCAGTCACTGG	TTGTGGAGCAGGTCAGGTTCAC
BTN2A1	TCTTCATGGTCACCACGGCTGT	ACAGGGAGACACACTGGGCATA
TL1A	CACCACATACCTGCTTGTCAGC	TCTCCGTCTGCTCTAAGAGGTG
CD137L	GGCTGGAGTCTACTATGTCTTCT	ACCTCGGTGAAGGGAGTCC
CD80	AAACTCGCATCTACTGGCAAA	GGTTCTTGTACTCGGGCCATA
HVEM	GTGCAGTCCAGGTTATCGTGT	CACTTGCTTAGGCCATTGAGG
CD48	GGCAGGGTCAGACTTGATCC	GTAGGTGCTGTTGTCCTCTTTC
B7H7	TACAAAGGCAGTGACCATTTGG	AGGTGTAAATTCCTTCGTCCAGA
VISTA	AGATGCACCATCCAACTGTGTGG	AGGCAGAGGATTCCTACGATGC
IDO	GCCTGATCTCATAGAGTCTGGC	TGCATCCCAGAACTAGACGTGC
TDO	TACCCTGCCCGAGTCTTCTG	TGGTGGTAGGTGTAGGGGTTC
SIGLECT15	CGCGGATCGTCAACATCTC	GTTCGGCGGTCACTAGGTG
CD252	CCAGGCCAAGATTCGAGAGG	CCGATGTGATACCTGAAGAGCA
GITRL	AGTGGCTCCCAATGCAAACTA	TATACAGCCGCACCTCAAAAG
NR3C1	TGCCGCTATCGAAAATGTCTT	GGGTAGGGGTGAGTTGTGGT

For mouse genes:

Gene name	Forward primer (5' to 3')	Reverse primer (5' to 3')
Cd274	TGCGGACTACAAGCGAATCACG	CTCAGCTTCTGGATAACCCTCG
B2m	ACAGTTCCACCCGCCTCACATT	TAGAAAGACCAGTCCTTGCTGAAG
H-2k	GGCAATGAGCAGAGTTTCCGAG	CCACTTCACAGCCAGAGATCAC
H-2d	TGAGGAACCTGCTCGGCTACTA	GGTCTTCGTTCAGGGCGATGTA
Fn1	CCCTATCTCTGATACCGTTGTCC	TGCCGCAACTACTGTGATTCGG
Klf9	CTACAGTGGCTGTGGGAAAGTC	CTCATCCGAGCGCGAGAACTTT
Snai2	TCTGTGGCAAGGCTTTCTCCAG	TGCAGATGTGCCCTCAGGTTTG
Nr3c1	CCGGGTCCCCAGGTAAAGA	TGTCCGGTAAAATAAGAGGCTTG
Gapdh	AGGTCGGTGTGAACGGATTTG	TGTAGACCATGTAGTTGAGGTCA

Supplementary Table 6. Primers for ChIP-qPCR

Gene name	Primer pair	Range	Forward primer (5' to 3')	Reverse primer (5' to 3')
PD-L1	#1	-1057 ~ -907	CCAATGCAAGGGCTATCTCAA	CACCGTGCCTGTGTGC
PD-L1	#2	-931 ~ -813	AAAAGGGAGCACACAGGCAC	AGTATTATCCCGCGCTGAAC
PD-L1	#3	-837 ~ -709	AGAAGTTCAGCGCGGGATAA	GGCTGCGGAAGCCTATTCT
PD-L1	#4	-663 ~ -569	AAGATGTAGCTCGGGATGGGA	ATCGTGGATTCTGTGACTTCC
PD-L1	#5	-590 ~ -474	GGAAGTCACAGAATCCACGA	AAAGTCAGCAGCAGACCCAT
PD-L1	#6	-335 ~ -237	GTGCGTTCAGATGTTGGCTT	ATTTTCACCGGGAAGAGTTTCG
PD-L1	#7	-148 ~ -65	CTAAACTGAAAGCTTCCGCCG	CTCTGCCCAAGGCAGCAAATC
HLA-A	#1	-980 ~ -884	TGAGGGGTTTCCTCCACCAT	CTCGGCCTCCCAAAGTGT
HLA-A	#2	-700 ~ -600	GCCACTGTACCCCAGCCT	GCTCACCAGCGTTCTTTCCT
HLA-A	#3	-481 ~ -374	CAAAATGAAAACACAGCTTTTGG	GGCAAAACAACACACATTTATCT
HLA-A	#4	-271 ~ -174	TGTGATGCAATTTAATACACTC	ACTTGAGCATATGAGGGGAT
HLA-B	#1	-913 ~ -792	ATTGGGACTGCATGGAGCACT	TTTCCCTGTGTGAGTCCAGAAC
HLA-B	#2	-746 ~ -667	TACAGATCCACAGTCTGGGAAA	AAAAGACCTGTGTCAAAACAGCAT
HLA-B	#3	-689 ~ -573	GCTGTTTTGACACAGGTCTTTTAC	AGGGATTCTCATAGAGACCAGTT
HLA-B	#4	-339 ~ -258	CTGGTTTCCACAGACAGATCCT	TGATCCCTCTTCTCCTACACCA
HLA-C	#1	-979 ~ -859	AAGAGCATTGGGACTGCATGG	GTGTGAGTCCAGAACATCTCC
HLA-C	#2	-815 ~ -717	ACCCAGAGTCACAGAACCAT	ATTCCAATCTGTGAAAGACCTGTG
HLA-C	#3	-699 ~ -586	ACCAATATTGTGCTACCTACTGT	AGGAGTTTTCTCTTTAAACCTGGTG
HLA-C	#4	-511 ~ -400	AGTCCAAGGGGAGAGGTAAGTTT	GTCCCTGAACTGGACTCCCTG
HLA-C	#5	-354 ~ -257	TTGGGGTCTCTCCCTGGTTTC	TCCTGATCCCTCTTCTCCTACA
B2M	#1	-982 ~ -864	ACTAAAATTGCCGAGCCCTTTG	CCATTCATTTTATGGACAGGGGA
B2M	#2	-612 ~ -537	ACAGCAAGGACATAGGGAGG	TGCTTCTTGTATCCCTTCAGGA
B2M	#3	-518 ~ -448	AGGACCTTCTCTGAGCTGTC	GGGTTTCTCCATTCTCTGGGT
B2M	#4	-419 ~ -324	ACAGAAGTTCTCCTTCTGCTAGG	AGTTAACTGCTCGGACCTGC
B2M	#5	-347 ~ -238	AATGCAGGTCCGAGCAGTTA	TCAGGCTGGAGGCACATTAAG

Supplementary Table 7. Mouse chemokine array coordinates

Coordinate	Analyte/Control	Entrez Gene ID	Alternate Nomenclature
A1, A2, A19,	Reference Spots	N/A	
B3, B4	6Ckine	18829	CCL21, SLC, Exodus-2
B5, B6	BLC	55985	CXCL13, BCA-1
B7, B8	C10	20305	CCL6, MRP-1
B9, B10	Complement Component C5/C5a	15139	C5/C5a
B11, B12	CCL28	56838	MEC
B13, B14	Chemerin	71660	RARRES2
B15, B16	CTACK	20301	CCL27, ALP, ILC, ESkine
B17, B18	CXCL16	66102	SRPSOX
C3, C4	Eotaxin	20292	CCL11
C5, C6	Fractalkine	20312	CX3CL1
C7, C8	IL-16	16170	
C9, C10	IP-10	15945	CXCL10, CRG-2, C7
C11, C12	I-TAC	56066	CXCL11, H174, SCYB9B
C13, C14	JE	20296	CCL2, MCP-1
C15, C16	KC	14825	CXCL1
C17, C18	LIX	20311	GCP-2, ENA-78
D3, D4	MCP-2	20307	CCL8, HC14
D5, D6	MCP-5	20293	CCL12
D7, D8	MDC	20299	CCL22, ABCD-1
D9, D10	MIG	17329	CXCL9, CRG-10, CMK
D11, D12	MIP-1α/β (pan)	20302/20303	CCL3/CCL4
D13, D14	MIP-1γ	20308	CCL9/10, CCF18, MRP-2
D15, D16	MIP-2	20310	CXCL2, GROβ, GRO2,
D17, D18	RANTES	20304	CCL5, SISd
E3, E4	SDF-1	20315	CXCL12, PBSF
E5, E6	Complement Factor D (Sample	11537	Adipsin, DF, Adn
E7, E8	gp130 (Sample Control)	16195	IL-6ST, CD130
E9, E10	HSP60 (Sample Control)	15510	Hspd1
E11, E12	Negative Control	N/A	
F1, F2	Reference Spots	N/A	
