# Supplementary material for: Glucocorticoid receptor regulates PD-L1 and MHC-I in pancreatic cancer cells to promote immune evasion and immunotherapy resistance
Source: Nat Commun. 2021 Dec 6;12:7041. doi: 10.1038/s41467-021-27349-7 (PMC8649069; doi:10.1038/s41467-021-27349-7)
Supplement: Supplementary file 2 — Reporting Summary [file 41467_2021_27349_MOESM2_ESM.pdf]

nature portfolio

Corresponding author(s): Li Ma
Last updated by author(s): Oct 20, 2021

Reporting Summary

Nature Portfolio wishes to improve the reproducibility of the work that we publish. This form provides structure for consistency and transparency in reporting. For further information on Nature Portfolio policies, see our Editorial Policies and the Editorial Policy Checklist.

Statistics

For all statistical analyses, confirm that the following items are present in the figure legend, table legend, main text, or Methods section.

n/a	Confirmed	
☐	☒	The exact sample size (*n*) for each experimental group/condition, given as a discrete number and unit of measurement
☐	☒	A statement on whether measurements were taken from distinct samples or whether the same sample was measured repeatedly
☐	☒	The statistical test(s) used AND whether they are one- or two-sided<br>*Only common tests should be described solely by name; describe more complex techniques in the Methods section.*
☒	☐	A description of all covariates tested
☐	☒	A description of any assumptions or corrections, such as tests of normality and adjustment for multiple comparisons
☐	☒	A full description of the statistical parameters including central tendency (e.g. means) or other basic estimates (e.g. regression coefficient) AND variation (e.g. standard deviation) or associated estimates of uncertainty (e.g. confidence intervals)
☐	☒	For null hypothesis testing, the test statistic (e.g. *F*, *t*, *r*) with confidence intervals, effect sizes, degrees of freedom and *P* value noted<br>*Give P values as exact values whenever suitable.*
☒	☐	For Bayesian analysis, information on the choice of priors and Markov chain Monte Carlo settings
☒	☐	For hierarchical and complex designs, identification of the appropriate level for tests and full reporting of outcomes
☐	☒	Estimates of effect sizes (e.g. Cohen's *d*, Pearson's *r*), indicating how they were calculated

Our web collection on statistics for biologists contains articles on many of the points above.

Software and code

Policy information about availability of computer code

Data collection	Flow cytometry data were collected using the Invitrogen Attune NxT Acoustic Focusing Cytometer. Quantitative PCR data were obtained with the Bio-Rad CFX96 Real-Time System. Fluorescent images were obtained using the Vectra Polaris Automated Quantitative Pathology Imaging System. Immunoblotting images were obtained using the ChemiDoc Touch Imaging System (Bio-Rad) and Image Lab Touch software (Bio-Rad, version 2.3.0.07). IHC images were obtained using a fully automated digital pathology slide-system (KFBIO, KF-PRO-005).
Data analysis	Flow cytometry data were analysed using FlowJo (FlowJo, version 10.4). Immunofluorescent images were analyzed using ImageJ (version 1.52p) or Phenochart (version 1.0.12). Statistical analysis was performed using Graphpad Prism (GraphPad software, version 8). CyTOF data were analyzed using Cytobank (https://premium.cytobank.org/cytobank/login). The correlation of expression levels of two genes was analyzed using the R corrplot package and the cor function. Survival analysis was performed using the R survival package.

For manuscripts utilizing custom algorithms or software that are central to the research but not yet described in published literature, software must be made available to editors and reviewers. We strongly encourage code deposition in a community repository (e.g. GitHub). See the Nature Portfolio guidelines for submitting code & software for further information.

Data

Policy information about availability of data

All manuscripts must include a data availability statement. This statement should provide the following information, where applicable:

- Accession codes, unique identifiers, or web links for publicly available datasets
- A description of any restrictions on data availability
- For clinical datasets or third party data, please ensure that the statement adheres to our policy

GR (encoded by NR3C1) mRNA levels in paired normal pancreatic tissue and PDAC were obtained from the dataset GSE15471 in the Gene Expression Omnibus

(https://www.ncbi.nlm.nih.gov/geo/query/acc.cgi?acc=GSE15471). TCGA gene expression data were obtained from The Cancer Genome Atlas data portal (https://tcga-data.nci.nih.gov/tcga/dataAccessMatrix.htm). The source data that support the findings of this study are available with no restrictions. The uncropped blots are shown in Supplementary Fig. 9. Source data are provided with this paper.

Field-specific reporting

Please select the one below that is the best fit for your research. If you are not sure, read the appropriate sections before making your selection.

☒ Life sciences ☐ Behavioural & social sciences ☐ Ecological, evolutionary & environmental sciences

For a reference copy of the document with all sections, see nature.com/documents/nr-reporting-summary-flat.pdf

Life sciences study design

All studies must disclose on these points even when the disclosure is negative.

Sample size	No sample size calculation was done either for in vitro or in vivo studies. For in vivo and in vitro studies, sample sizes were determined based on our preliminary experiments. In our experience, n = 5-7 mice per group (in vivo) and n = 3-4 samples per group (in vitro) are sufficient to detect meaningful biological differences with good reproducibility.
Data exclusions	No data or animals were excluded from analysis.
Replication	Except for the animal studies (one time), chemokine array analysis (one time), and tissue microarray analysis (one time), each experiment was repeated at least three times with similar results.
Randomization	Mice were randomly assigned to different treatment groups.
Blinding	For cell-based experiments, Western blotting, flow cytometry, and in vitro assays, blinding was not performed, because the investigator had to know the groups to load the samples or perform the assay. Blinding was not performed in mouse experiments. The investigator needed to know the treatment groups in order to perform the study.

Reporting for specific materials, systems and methods

We require information from authors about some types of materials, experimental systems and methods used in many studies. Here, indicate whether each material, system or method listed is relevant to your study. If you are not sure if a list item applies to your research, read the appropriate section before selecting a response.

Materials & experimental systems

n/a	Involved in the study	
☐	☒	Antibodies
☐	☒	Eukaryotic cell lines
☒	☐	Palaeontology and archaeology
☐	☒	Animals and other organisms
☐	☒	Human research participants
☒	☐	Clinical data
☒	☐	Dual use research of concern

Methods

n/a	Involved in the study	
☒	☐	ChIP-seq
☐	☒	Flow cytometry
☒	☐	MRI-based neuroimaging

Antibodies

Antibodies used

Antibodies used for mouse experiments:
anti-mouse CD8α antibody (200 μg, clone 2.43, BE0061, Bio X Cell; RRID: AB_1125541)
rat IgG2b isotype control (200 μg, LTF-2, BE0090, Bio X Cell; RRID: AB_1107780)
anti-mouse PD-1 antibody (100 μg, clone RMP1-14, BP0146, Bio X Cell; RRID: AB_10949053)
anti-mouse CTLA-4 antibody (100 μg, clone UC10-4F10-11, BE0032, Bio X Cell; RRID: AB_1107598)
rat IgG2a isotype control (100 μg, clone 2A3, BE0089, Bio X Cell; RRID: AB_1107769)
hamster IgG (100 μg, Bio X Cell, BE0091; RRID: AB_1107773)

Antibodies used for immunofluorescent staining:
CD3 (1:200, Cell Signaling Technology, 99940S, RRID: AB_2755035)
CD8 (1:200, Cell Signaling Technology, 98941S, RRID: AB_2756376)
Granzyme B (1:200, Cell Signaling Technology, 44153S, RRID: AB_2857976)

Antibodies used for IHC:
GR (1:200, Sigma-Aldrich, SAB4501309, RRID: AB_10744954)
PD-L1(1:200, GeneTex, GTX01796)
MHC-I (1:200, Santa Cruz, sc55582, RRID: AB_831547)

CD8 (1:100, MXB Biotechnologies, RMA-0514)

Antibodies used for immunoblotting:
GR (1:1000, Proteintech, 24050-1-AP, RRID:AB_2813890)
Phospho-GR (Ser211) (1:1000, Cell Signaling Technology, 4161S, RRID:AB_2155797)
GAPDH (1:2000, Proteintech, 60004-1-IG, RRID:AB_2107436)
PD-L1 (1:1000, Cell Signaling Technology, 13684S, RRID:AB_2687655)
MHC-I (1:500, Santa Cruz Biotechnology, sc-55582, RRID:AB_831547)
MHC-I (1:500, Santa Cruz Biotechnology, sc-32235, RRID:AB_627934)
B2M (1:1000, Cell Signaling Technology, 12851S, RRID:AB_2716551)
Phospho-STAT1 (1:1000, Cell Signaling Technology, 9167S, RRID:AB_561284)
STAT1 (1:1000, Cell Signaling Technology, 14994S, RRID:AB_2737027)
PR (1:1,000, Proteintech, 25871-1-AP, RRID:AB_2880277)

Antibodies used for flow cytometry:
PD-L1: APC, 10F.9G2, 1:80, BioLegend, 124312
CD45: PE, 30-F11, 1:100, BioLegend, 103106
CD45: FITC, 30-F11, 1:100, BioLegend, 103108
CD3: APC, 145-2C11, 1:40, BioLegend, 100312
CD8: APC-Cy7, 53-6.7, 1:40, BioLegend, 100714
CD4: FITC, GK1.5, 1:80, BioLegend, 100406.
B2M: APC, A16041A, 1:40, BioLegend, 154506
B2M: PE, A16041A, 1:40, BioLegend, 156504
H-2Kb: PE-Cy7, AF6-88.5, 1:80, BioLegend, 116520
H-2Kb: APC, AF6-88.5, 1:80, BioLegend, 116518
H-2Kb/Db: 28-8-6, 1:200, BioLegend, 114602
Luciferase: PE, Luci 21 1-107, 1:200, Novus Biologicals, NB600-307PE
HLA-A/B/C: PE, W6/32, 1:40, BioLegend, 311406
HLA-A/B/C: PE-Cy5, G46-2.6, 1:10, BD, 555554
B2M: FITC, 2M2, 1:40, BioLegend, 316304
PD-L1: APC, 29E.2A3, 1:40, BioLegend, 329708
IL-2: PE, JES6-5H4, 1:40, BioLegend, 503807
TNFα: PE, MP6-XT22, 1:80, BioLegend, 506305
IFNγ: PE-Cy7, XMG1.2, 1:40, BioLegend, 505825
PD-1: PE-Cy7, 29F.1A12, 1:80, BioLegend, 135215
LAG-3: PE, C9B7W, 1:40, BioLegend, 125207
Tim-3: PE, RMT3-23, 1:80, BioLegend, 119703

Antibodies used for CyTOF analysis
For HY24409 tumors:
CD11b 139La M1/70 1:500 BioLegend 101249
CD11c 142Nd N418 1:400 BioLegend 117302
CD19 149Sm 4D5 1:400 BioLegend 115502
CD25 150Nd 3C7 1:200 BioLegend 101902
CD3, CD3e 152Sm 145-2C11 1:500 BioLegend 100302
CD4(Ms) 115In RM4-5 1:500 BioLegend 100506
CD45(Ms) 89Y 30-F11 1:400 DVS-Fluidigm 3089005B
CD8a 146Nd 53-6.7 1:500 BioLegend 100702
F4/80 171Yb D2S9R 1:400 CST 70076BF
Foxp3 158Gd FJK-16s 1:500 DVS-Fluidigm 3158003A
I-A/I-E, MHC-II 209Bi M5/114.15.2 1:1000 DVS-Fluidigm 3209006B
Ly-6G/Ly-6C, Gr-1 141Pr RB6-8C5 1:800 BioLegend 108402
NK1.1 170Er PK136 1:400 BioLegend 108702
For HY24160 tumors:
CD45(Ms) 89Y 30-F11 1:400 DVS-Fluidigm 3089005B
CD3, CD3e 152Sm 145-2C11 1:500 BioLegend 100302
CD8a 146Nd 53-6.7 1:500 BioLegend 100702
CD4(Ms) 115In RM4-5 1:500 BioLegend 100506
F4/80 173Yb BM8 1:400 BioLegend 123102
CD11b 139La M1/70 1:500 BioLegend 101249
Ly-6C 162Dy HK1.4 1:600 DVS-Fluidigm 3162014B
Ly-6G 141Pr 1A8 1:400 DVS-Fluidigm 3141008B
CD25 150Nd 3C7 1:200 BioLegend 101902
Foxp3 158Gd FJK-16s 1:500 DVS-Fluidigm 3158003A
CD19 149Sm 4D5 1:400 BioLegend 115502
CD11c 142Nd N418 1:400 BioLegend 117302
NK1.1 170Er PK136 1:400 BioLegend 108702
I-A/I-E, MHC-II 209Bi M5/114.15.2 1:1000 DVS-Fluidigm 3209006B

Validation

All antibodies used are commercially available and validated by the manufacturers. Pre-validated antibodies were purchased from reputable sources. All proteins are well studied and all antibodies are widely used in the literature. The experiments included appropriate controls. We validated the GR-specific antibody by using two independent GR shRNAs.

Antibodies used for mouse experiments:
anti-mouse CD8α antibody (BE0061, Bio X Cell; RRID: AB_1125541) https://bxcell.com/product/m-cd8a-2/
rat IgG2b isotype control (BE0090, Bio X Cell; RRID: AB_1107780) https://bxcell.com/product/rat-igg2b-isotype-control/

anti-mouse PD-1 antibody (BP0146, Bio X Cell; RRID: AB_10949053) https://bxcell.com/product/invivoplus-anti-m-pd-1/
anti-mouse CTLA-4 antibody (BE0032, Bio X Cell; RRID: AB_1107598) https://bxcell.com/product/m-cd152-m-ctla-4-2/
rat IgG2a isotype control (BE0089, Bio X Cell; RRID: AB_1107769) https://bxcell.com/product/rat-igg2a-isotype-control/
hamster IgG (BE0091, Bio X Cell; RRID: AB_1107773) https://bxcell.com/product/polyclonal-3/

Antibodies used for immunofluorescent staining:
CD3 (Cell Signaling Technology, 99940S, RRID: AB_2755035) https://www.cellsignal.com/products/primary-antibodies/cd3e-d4v8l-rabbit-mab/99940
CD8 (Cell Signaling Technology, 98941S, RRID: AB_2756376) https://www.cellsignal.com/products/primary-antibodies/cd8a-d4w2z-xp-rabbit-mab-mouse-specific/98941
Granzyme B (Cell Signaling Technology, 44153S, RRID: AB_2857976) https://www.cellsignal.com/products/primary-antibodies/granzyme-b-e5v2l-rabbit-mab-mouse-specific/44153

Antibodies used for IHC:
GR (Sigma-Aldrich, SAB4501309, RRID: AB_10744954) https://www.sigmaaldrich.com/US/en/product/sigma/sab4501309
PD-L1(GeneTex, GTX01796) https://www.genetex.com/Product/Detail/PD-L1-antibody-ZR3/GTX01796
MHC-I (Santa Cruz, sc55582, RRID: AB_831547) https://www.scbt.com/p/mhc-class-i-antibody-f-3
CD8 (MXB Biotechnologies, RMA-0514) http://maxim.com.cn/sitecn/dklkthdklkt/7013.html

Antibodies used for immunoblotting:
GR (Proteintech, 24050-1-AP, RRID:AB_2813890) https://www.ptglab.com/products/NR3C1-Antibody-24050-1-AP.htm
Phospho-GR (Ser211) (Cell Signaling Technology, 4161S, RRID:AB_2155797) https://www.cellsignal.com/products/primary-antibodies/phospho-glucocorticoid-receptor-ser211-antibody/4161
GAPDH (Proteintech, 60004-1-IG, RRID:AB_2107436) https://www.ptglab.com/products/GAPDH-Antibody-60004-1-Ig.htm
PD-L1 (Cell Signaling Technology, 13684S, RRID:AB_2687655) https://www.cellsignal.com/products/primary-antibodies/pd-l1-e1l3n-xp-rabbit-mab/13684
MHC-I (Santa Cruz Biotechnology, sc-55582, RRID:AB_831547) https://www.scbt.com/p/mhc-class-i-antibody-f-3
MHC-I (Santa Cruz Biotechnology, sc-32235, RRID:AB_627934) https://www.scbt.com/p/mhc-class-i-antibody-w6-32
B2M (Cell Signaling Technology, 12851S, RRID:AB_2716551) https://www.cellsignal.com/products/primary-antibodies/b2-microglobulin-d8p1h-rabbit-mab/12851
Phospho-STAT1 (Cell Signaling Technology, 9167S, RRID:AB_561284) https://www.cellsignal.com/products/primary-antibodies/phospho-stat1-tyr701-58d6-rabbit-mab/9167
STAT1 (Cell Signaling Technology, 14994S, RRID:AB_2737027) https://www.cellsignal.com/products/primary-antibodies/stat1-d1k9y-rabbit-mab/14994
PR (Proteintech, 25871-1-AP, RRID:AB_2880277) https://www.ptglab.com/products/PR-Antibody-25871-1-AP.htm

Antibodies used for flow cytometry:
PD-L1: BioLegend, 124312 https://www.biolegend.com/en-us/products/apc-anti-mouse-cd274-b7-h1-pd-l1-antibody-6655?GroupID=BLG5396
CD45: BioLegend, 103106 https://www.biolegend.com/en-us/products/pe-anti-mouse-cd45-antibody-100
CD45: BioLegend, 103108 https://www.biolegend.com/en-us/products/fitc-anti-mouse-cd45-antibody-99
CD3: BioLegend, 100312 https://www.biolegend.com/en-us/products/apc-anti-mouse-cd3epsilon-antibody-21
CD8: BioLegend, 100714 https://www.biolegend.com/en-us/products/apc-cyanine7-anti-mouse-cd8a-antibody-2269
CD4: BioLegend, 100406 https://www.biolegend.com/en-us/products/fitc-anti-mouse-cd4-antibody-248
B2M: BioLegend, 154506 https://www.biolegend.com/en-us/products/apc-anti-mouse-beta2-microglobulin-antibody-15125
B2M: BioLegend, 154504 https://www.biolegend.com/en-us/products/pe-anti-mouse-beta2-microglobulin-antibody-15126
H-2Kb: BioLegend, 116520 https://www.biolegend.com/en-us/products/pe-cyanine7-anti-mouse-h-2kb-antibody-15167
H-2Kb: BioLegend, 116518 https://www.biolegend.com/en-us/products/apc-anti-mouse-h-2kb-antibody-6573
H-2Kb/Db: BioLegend, 114602 https://www.biolegend.com/en-us/products/purified-anti-mouse-h-2k-b-h-2d-b-antibody-1684
Luciferase: Novus Biologicals, NB600-307PE https://www.novusbio.com/products/luciferase-antibody-luci-21-1-107_nb600-307pe
HLA-A/B/C: BioLegend, 311406 https://www.biolegend.com/en-us/products/pe-anti-human-hla-a-b-c-antibody-1872
HLA-A/B/C: BD, 555554 https://www.bdbiosciences.com/en-us/products/reagents/flow-cytometry-reagents/research-reagents/single-color-antibodies-ruo/pe-cy-5-mouse-anti-human-hla-abc.555554
B2M: BioLegend, 316304 https://www.biolegend.com/en-us/products/fitc-anti-human-beta2-microglobulin-antibody-3079
PD-L1: BioLegend, 329708 https://www.biolegend.com/en-us/products/apc-anti-human-cd274-b7-h1-pd-l1-antibody-4376
IL-2: BioLegend, 503807 https://www.biolegend.com/en-us/products/pe-anti-mouse-il-2-antibody-954
TNFα: BioLegend, 506305 https://www.biolegend.com/en-us/products/pe-anti-mouse-tnf-alpha-antibody-978
IFNγ: BioLegend, 505825 https://www.biolegend.com/en-us/products/pe-cyanine7-anti-mouse-ifn-gamma-antibody-5865
PD-1: BioLegend, 135215 https://www.biolegend.com/en-us/products/pe-cyanine7-anti-mouse-cd279-pd-1-antibody-7005
LAG-3: BioLegend, 125207 https://www.biolegend.com/en-us/products/pe-anti-mouse-cd223-lag-3-antibody-4486
Tim-3: BioLegend, 119703 https://www.biolegend.com/en-us/products/pe-anti-mouse-cd366-tim-3-antibody-2657

Antibodies used for CyTOF analysis
CD11b BioLegend 101249 https://www.biolegend.com/en-us/products/purified-anti-mouse-human-cd11b-maxpar-ready-antibody-9159
CD11c BioLegend 117302 https://www.biolegend.com/en-us/products/purified-anti-mouse-cd11c-antibody-1817
CD19 BioLegend 115502 https://www.biolegend.com/en-us/products/purified-anti-mouse-cd19-antibody-1532
CD25 BioLegend 101902 https://www.biolegend.com/en-us/products/purified-anti-mouse-cd25-antibody-130
CD3, CD3e BioLegend 100302 https://www.biolegend.com/en-us/products/purified-anti-mouse-cd3epsilon-antibody-28
CD4(Ms) BioLegend 100506 https://www.biolegend.com/en-us/products/purified-anti-mouse-cd4-antibody-484
CD45(Ms) DVS-Fluidigm 3089005B https://store.fluidigm.com/Cytometry/ConsumablesandReagentsCytometry/MaxparAntibodies/Anti-Mouse%20CD45%20-30-F11-89Y%E2%80%94100%20Tests?cclcl=en_US
CD8a BioLegend 100702 https://www.biolegend.com/en-us/products/purified-anti-mouse-cd8a-antibody-157
F4/80 Cell Signaling Technology 70076BF https://www.cellsignal.com/products/primary-antibodies/f4-80-d2s9r-xp-rabbit-mab/70076
Foxp3 DVS-Fluidigm 3158003A https://store.fluidigm.com/Cytometry/ConsumablesandReagentsCytometry/MaxparAntibodies/Anti-Mouse%20Foxp3%20-FJK-16s-158Gd%E2%80%9450%20Tests?cclcl=en_US

I-A/I-E, MHC-II DVS-Fluidigm 3209006B https://store.fluidigm.com/Cytometry/ConsumablesandReagentsCytometry/MaxparAntibodies/Anti-Mouse%20I-A-I-E%20-M5-114-15-2-209Bi%E2%80%94100%20Tests
Ly-6G/Ly-6C, Gr-1 BioLegend 108402 https://www.biolegend.com/en-us/products/purified-anti-mouse-ly-6g-ly-6c-gr-1-antibody-462
NK1.1 BioLegend 108702 https://www.biolegend.com/en-us/products/purified-anti-mouse-nk-1-1-antibody-432
F4/80 BioLegend 123102 https://www.biolegend.com/en-us/products/purified-anti-mouse-f4-80-antibody-4064
Ly-6C DVS-Fluidigm 3162014B https://store.fluidigm.com/Cytometry/ConsumablesandReagentsCytometry/MaxparAntibodies/Anti-Mouse%20Ly-6C%20-HK1-4-162Dy%E2%80%94100%20Tests?cclcl=en_US
Ly-6G DVS-Fluidigm 3141008B https://store.fluidigm.com/Cytometry/ConsumablesandReagentsCytometry/MaxparAntibodies/Anti-Mouse%20Ly-6G%20-1A8-141Pr%E2%80%94100%20Tests?cclcl=en_US

Eukaryotic cell lines

Policy information about cell lines

Cell line source(s)	The BXPC-3 (ATCC), Miapaca-2(ATCC), Capan-1(ATCC), ASPC-1(ATCC), Colo357/FG, L3.6PL, Panc28, Capan-2(ATCC), CF-Pac-1(ATCC), Panc3, Panc1(ATCC), Panc48, HPAC(ATCC), Hs766T(ATCC), SU86.86(ATCC), and SW1990 (ATCC) cell lines were from Dr. Mien-Chie Hung's lab stock. HY24409, HY19636, and HY24160 cell lines were from Dr. Haoqiang Ying. MCF-7 and HEK293T were purchased from ATCC.
Authentication	Short tandem repeat (STR) profiling was done by ATCC and MD Anderson’s Characterized Cell Line Core Facility.
Mycoplasma contamination	All cell lines were confirmed to be mycoplasma free with a mycoplasma detection kit and treated with Plasmocin for the Prevention of mycoplasma contamination.
Commonly misidentified lines (See ICLAC register)	No commonly misidentified cell lines were used.

Animals and other organisms

Policy information about studies involving animals; ARRIVE guidelines recommended for reporting animal research

Laboratory animals	Male and female C57BL/6 mice were from MD Anderson’s internal supply or the Jackson Laboratory, and we performed the surgery when the mice were 6-8 weeks old. Male NSG (non-obese diabetic; severe combined immunodeficiency; interleukin-2 receptor gamma chain null) mice were from MD Anderson’s internal supply, and six-week-old NSG mice received subcutaneous injection of tumor cells.<br><br>Mice were housed at 70F-74F (set point: 72F) with 40%-55% humidity (set point: 45%). The light cycle of animal rooms is 12 h of light and 12 h of dark.
Wild animals	This study did not involve wild animals.
Field-collected samples	This study did not involve samples collected in the field.
Ethics oversight	All animal studies were performed in accordance with a protocol (PI: Li Ma) approved by the Institutional Animal Care and Use Committee of MD Anderson Cancer Center.

Note that full information on the approval of the study protocol must also be provided in the manuscript.

Human research participants

Policy information about studies involving human research participants

Population characteristics	Our research did not involve human subjects, but used human samples from the Cancer Hospital of the University of Chinese Academy of Sciences. All PDAC specimens were obtained with informed consent from patients who underwent surgical resection of primary tumors. They were de-identified specimens.
Recruitment	We did not recruit patients.
Ethics oversight	The collection and use of human samples were approved by the Ethics Committee of Cancer Hospital of the University of Chinese Academy of Sciences, following the Declaration of Helsinki ethical guidelines.

Note that full information on the approval of the study protocol must also be provided in the manuscript.

Flow Cytometry

Plots

Confirm that:

☒ The axis labels state the marker and fluorochrome used (e.g. CD4-FITC).

☒ The axis scales are clearly visible. Include numbers along axes only for bottom left plot of group (a 'group' is an analysis of identical markers).

☒ All plots are contour plots with outliers or pseudocolor plots.

☒ A numerical value for number of cells or percentage (with statistics) is provided.

Methodology

Sample preparation	For cultured cell lines, cells were incubated with the Accutase Cell Detachment Solution (BioLegend, 423201) and were washed twice with PBS. For the immunophenotyping of tumors, tumor samples were dissociated on the gentleMACS Dissociator (Miltenyi Biotec) using the Mouse Tumor Dissociation Kit (Miltenyi Biotec, 130-096-730) and were depleted of red blood cells using RBC Lysis Buffer (BioLegend, 420301). 1 × 106 cells per sample were used for staining.
Instrument	Cells were analyzed on an Invitrogen Attune NxT Acoustic Focusing Cytometer.
Software	Cells were analyzed on an Invitrogen Attune NxT Acoustic Focusing Cytometer and analyzed by FlowJo software (FlowJo, LLC, version 10.4).
Cell population abundance	At least 10,000 cells were analyzed for each sample.
Gating strategy	Gating strategies are described in the Methods section (Flow cytometry) and Supplementary Table 2.

☒ Tick this box to confirm that a figure exemplifying the gating strategy is provided in the Supplementary Information.
